# From Light to Logic: Recent Advances in Optoelectronic Logic Gate

**DOI:** 10.1002/smsc.202400264

**Published:** 2024-11-03

**Authors:** Woochul Kim, Dante Ahn, Minz Lee, Namsoo Lim, Hyeonghun Kim, Yusin Pak

**Affiliations:** ^1^ Sensor System Research Center Korea Institute of Science and Technology (KIST) Seoul 02792 Republic of Korea; ^2^ Semiconductor R&D Center Samsung Electronics Hwaseong 18448 South Korea; ^3^ KU‐KIST Graduate School of Converging Science and Technology Korea University Seoul 02841 Republic of Korea; ^4^ Department of Materials Science and Engineering Korea University Seoul 02841 Republic of Korea; ^5^ Ceramic Total Solution Center Korea Institute of Ceramic Engineering and Technology Icheon Gyeonggi 17303 Republic of Korea; ^6^ School of Chemical Engineering Chonnam National University Gwangju 61186 Republic of Korea

**Keywords:** bipolar photoresponses, in‐memory computing, multifunctional logics, optoelectronic logic gates, reconfigurable logics

## Abstract

This review delves into the advancements in optoelectronic logic gate (OELG) devices, emphasizing their transformative potential in computational technology through the integration of optical and electronic components. OELGs present significant advantages over traditional electronic logic gates, including enhanced processing speed, bandwidth, and energy efficiency. The evolution of OELG architectures from single‐device, single‐logic systems to more sophisticated multidevice, multilogic, and reconfigurable OELGs is comprehensively explored. Key advancements include the development of materials and device structures enabling multifunctional logic operations and the incorporation of in‐memory functionalities, critical for applications in high‐performance computing and real‐time data processing. This review also addresses the challenges that need to be overcome, such as stability, durability, integration with existing semiconductor technologies, and efficiency. By summarizing current research and proposing future directions, this review aims to guide the ongoing development of next‐generation optoelectronic architectures, poised to redefine the landscape of optical computing, communication, and data processing.

## Graphical abstract

This review delves into advancements in optoelectronic logic gate (OELG) devices, emphasizing their transformative potential in computational technology through the integration of optical and electronic components. It highlights the evolution from single‐device, single‐logic systems to multidevice, multilogic, and in‐memory OELGs, driven by the need to enhance speed, bandwidth, and energy efficiency.

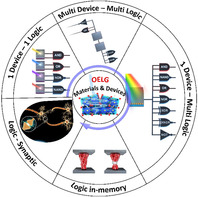

## 1 Introduction

Optoelectronic logic gates (OELGs) are pivotal devices that are revolutionizing the landscape of computational technology by executing logic operations through the interplay of light signals and electronic components.^[^ ^1^, ^2^ ^]^ Note that to avoid confusion with all‐optical logic gate (OLG), herein we suggest the term OELG instead of OLGD, which is commonly found in pertinent literature. These devices, which include fundamental elements such as photodiodes and phototransistors, convert optical inputs into digital signals, thereby facilitating high‐speed data processing and communication. The basic logic operations performed by OELGs—AND, OR, NOT, NAND, and NOR—serve as the building blocks of future integrated circuits in advanced computing systems.

**Scheme 1:**
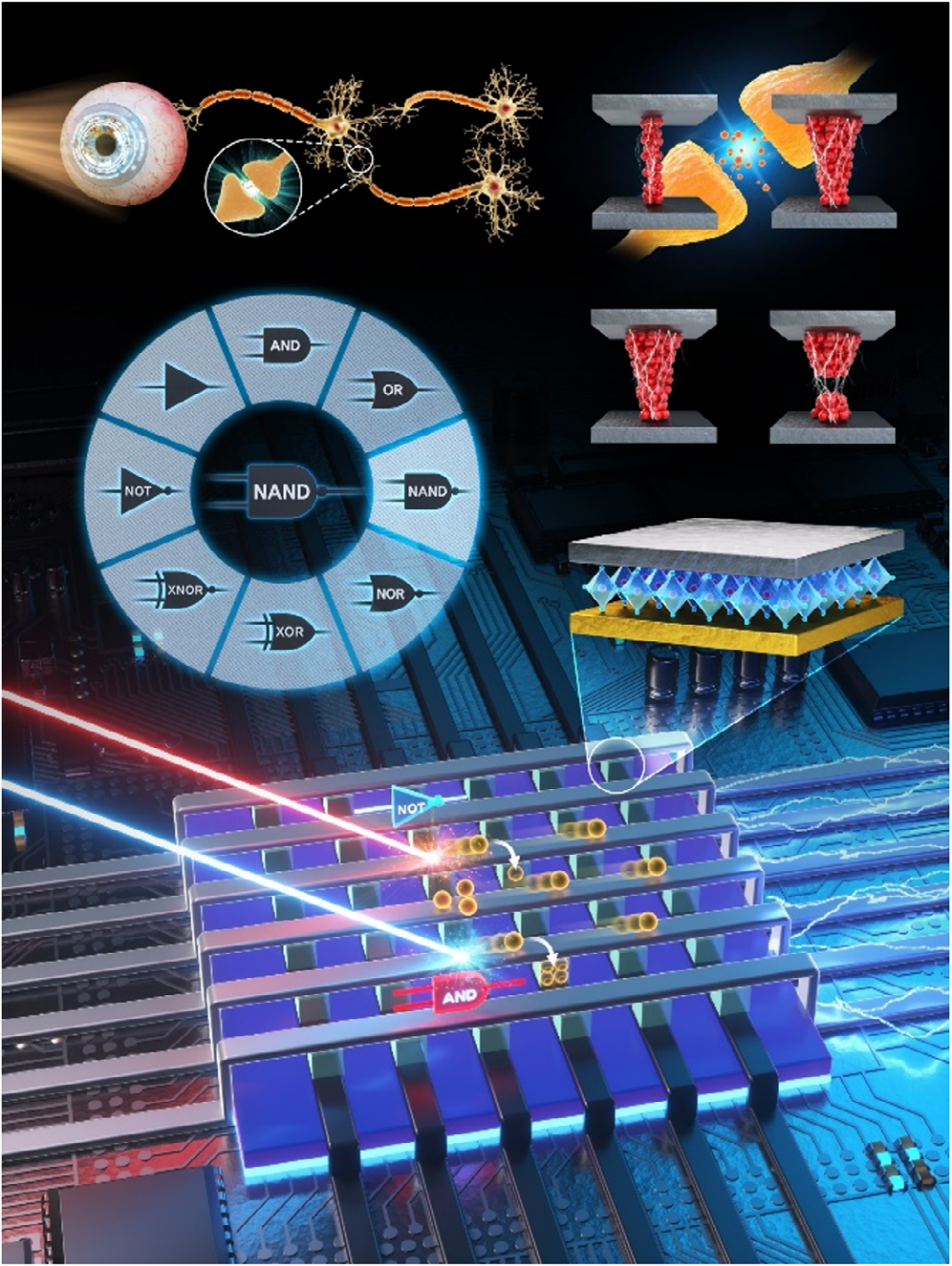
Schematic representation of various applications such as computing and memory devices utilizing optoelectronic materials (Original and created by the authors).

The allure of OELGs lies in their potential to surpass traditional silicon‐based logic devices, which are limited by their electronic nature and material constraints.^[^ ^3^ ^]^ Additionally, efforts to develop all‐OLGs, where both inputs and outputs are mediated through light, have faced challenges, particularly in achieving low optical losses and effective miniaturization.^[^ ^4^, ^5^ ^]^ Conversely, in OELGs, by integrating optoelectronic components that leverage light for information transfer, OELGs offer significant advantages such as lower power consumption, faster processing speeds, and enhanced bandwidth. This makes them highly suitable for applications in next‐generation optical computing, high‐performance computing, and real‐time data‐intensive tasks like Internet of Things and deep neural networks. Moreover, the incorporation of in‐memory functionalities allows these devices to perform logic operations while simultaneously managing data storage, a capability that is increasingly vital in fields such as database management, caching, and session storage (**Scheme** **1**).

**Figure 1:**
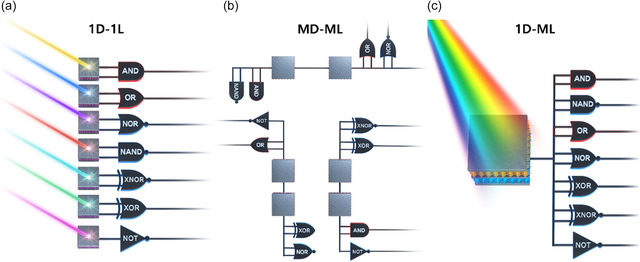
Development directions of OELG devices from a–c. a) Single device performing 1D‐1L. b) Integration of multiple devices for implementing MD‐ML. c) Single device used for 1D‐ML. (Original and created by the authors).

Instead of relying solely on electrical signals, OELG devices use light signals to process and manipulate information. This requires the use of optoelectronic devices, such as photodiodes, which convert light input into digital electronic signals to control the input and output of light under specific conditions. Early research into OELGs used device architectures such as PN, PIN, and avalanche photodiode, incorporating silicon nanowire (NW), compound semiconductors, and metal oxide materials.^[^ ^6^, ^7^, ^8^, ^9^ ^]^ However, these devices are based on unidirectional photocarrier transport, making them suitable only for single‐logic operation by single device (1D‐1L), while multiple‐logic operations are only possible through circuit layouts that combine multiple optoelectronic devices (MD‐ML), as shown in **Figure** **1**a,b. Research has evolved from 1D‐1L operations to MD‐ML systems. (**Table** **1**) Along with this progression, carbon allotropes,^[^ ^10^, ^11^ ^]^ organics,^[^ ^12^, ^13^ ^]^ and perovskites^[^ ^14^, ^15^ ^]^ were researched. These materials allow the simple and rapid design of heterojunctions, facilitating the engineering of photocarrier generation and transport properties.

**Table 1:** Types of input and output signals and implementable logic functions by OLEG device circuit structure.

Key function		Input method	Output method	Logic function	References
Logic	1D‐1L	Electric field	Optical	NOT, NOR, NAND	[4]
		Light	Optical	AND	[5]
		Light	Electrical	AND, OR, NAND	[6]
		Light	Optical	OR	[9]
		Light	Electrical	NOT	[21]
	MD‐ML	Light	Electrical	AND, OR	[18]
		Electrical & Light	Electrical	AND, OR	[76]
		Electrical & Light	Electrical	AND, Xf, OR	[75]
		Light	Electrical	AND, OR	[74]
	1D‐ML	Light	Electrical	AND, OR, NOT, NAND, NOR	[11]
		Light	Electrical	OR, AND	[12]
		Light	Electrical	AND, OR, NAND, NOR, NOT	[16]
		Light	Electrical	NOT, XOR, OR, ternary OR	[118]
		Light	Electrical	NOR, XOR, NAND	[24]

Recently, 1D‐ML have been investigated for improved energy and spatial efficiency, and research is expanding to multifunctional OELG devices with synaptic in‐memory properties,^[^ ^16^, ^17^ ^]^ as depicted in Figure 1c. When in‐memory functionality as well as logic operations are enabled by optical inputs, multiple current and voltage states can be dynamically programmed with light intensity, wavelength, or various optical properties, which holds great promise for applications in artificial intelligence learning and cognitive computing.

To implement 1D‐ML, it is necessary to develop materials or device structures with bipolar photoresponse (photogenerated current or voltage can have both positive and negative directions). Bipolar photoresponse has been achieved mainly by 1) heterojunction devices for wavelength‐dependent light absorption and photocarrier generation/separation; 2) stacked thin films with different photocarrier generation mechanisms; and 3) defect engineering techniques. The spectral range for optical input is constrained by the limited number of materials suitable for complex heterojunction and electrode processes. In addition, there is a need for multifaceted verification in terms of efficiency, durability, reliability, integration, and compatibility with existing electronic systems.

The aim of this review is to provide researchers in related fields with the right research direction by summarizing the existing research leading to 1D‐1L, MD‐ML, and 1D‐ML that have been reported to date. To facilitate the understanding of researchers in other fields or general readers, the methods of optoelectronic bit generation and the related photocarrier generation mechanisms are first explained in a simple way. Finally, in Discussion and Summary, we propose the design and development directions of optoelectronic architectures with 1D‐ML and in‐memory features suitable for future applications such as optoelectronic on‐chip interconnects, in‐memory computing, cognitive computing, and visual information processing, based on the conclusions and implications drawn from the analysis of existing research.

## 2 Photogeneration Method Depending on the Material

OELGs employ light as an input signal for the purpose of processing digital information. This device utilizes photocurrent to differentiate between digital bits 0 and 1, with the photocurrent varying in accordance with the intensity of light. Maintaining a constant dark current intensity in the dark and photocurrent intensity in the light is essential for accurate representation of digital bits. In addition, advanced materials are utilized to improve the functionality of the OELGs. Utilizing low‐dimensional materials, perovskites, carbon allotropes, and organic materials in novel structures enable enhanced modulation of photocurrent intensity, thereby enhancing reliability. Specifically, these materials have the ability to cause electrical hysteresis through modifying defects or ion concentration, thereby incorporating more complicated memory capabilities into devices.

OELGs may be classified according to the electrical and thermal states induced as a result of the physical changes in the molecules or crystals that comprise the light‐absorbing materials. Typically, the most extensively researched types are those that rely on the photovoltaic effect (PVE)^[^ ^18^, ^19^, ^20^ ^]^ and the photoconductive conversion efficiency (PCE).^[^ ^21^, ^22^, ^23^, ^24^ ^]^ The PVE is a phenomenon that produces a substantial electric current without the need for an external voltage. It achieves this utilizing an internal electric field that is influenced by light, effectively separating the charge carriers generated by light. The PCE effect arises from changes in electrical conductivity, which are linked to the concentration of trapped charges influenced by the surrounding illumination.

In this section, we discuss OELGs that operate on the basis of PVE and PCE, focusing on the most advanced and widely studied sensing materials to date. **Table** **2** summarizes the OELG research results, which were not covered in detail in the text, focusing on the subject matter. Comparing the advantages and disadvantages of each material in implementing logic operations yields the following insights. Organic–inorganic hybrid perovskites (OIHPs) offer high absorption coefficients and tunable bandgaps, enabling effective absorption of various wavelengths of light and cost‐efficient solution‐based manufacturing. However, they are susceptible to heat, light, and moisture, resulting in low stability and challenges in commercialization. Conjugated polymers can absorb a wide range of wavelengths and allow for flexible design with low power operation. Nevertheless, their low carrier mobility and sensitivity to environmental changes make self‐powered operation difficult. Single‐molecule devices (SMDs) offer the potential for miniaturization and high‐resolution analysis, but they have complex manufacturing processes and potentially low stability. Van der Waals (vdW) heterojunctions provide high mobility and precise logic definition, but they are sensitive to gases and moisture and face challenges in fabricating highly integrated circuits on a large scale. Crystalline silicon and inorganic materials can absorb a wide spectrum of light, offering high stability and performance, and are easily integrated with existing semiconductor technology, though they can be expensive to manufacture and lack flexibility. In conclusion, each material possesses unique strengths and weaknesses. When implementing logic operations, it is crucial to consider factors such as stability, performance, and manufacturability to select the most suitable material and structure for the specific application.

**Table 2:** Summary of mobility, response time, and power consumption of optoelectronic materials from cited studies. Only materials that served as the photoactive layer in the studied devices are listed. The ‘–' symbol indicates values that are ‘unspecified' or ‘not mentioned' in the references.

Material category	References	Material	Mobility [cm^2^ V^−1^ s^−1^]	Response time	On/off ratio	Power consumption
Semiconductor	[4]	CdS	–	3 μs	–	4.5 mW cm^−^^2^
	[8]	ZnO, CdSe/ZnS QDs	0.746, 0.02	–	107	4.5 mW cm^−2^
	[9]	CuInS_2_	–	2 ms	–	–
	[44]	GaN, RuO_x_	–	–	>10^6^	–
	[50]	Crystalline silicon	–	37–49 μs	105	≈2.5 fJ
	[109]	Ta_2_O_5_, Ta	–	50 ms–1 s	–	–
	[53]	Sb_2_Se_3_	–	43–87 ms	3100	25 mW cm^−^^2^
	[66]	GaInAsP	–	<1 ns	–	–
	[78]	CdTe/SnSe	–	20.9–32.6 ms	–	–
	[74]	GaN NW	–	<26 ms	–	1 mW cm^−^^2^
	[75]	ScN	20–120	–	–	0.13nW mm^−2^
Oxide	[13]	ZnO	–	110–200 ms	–	2.47 mW cm^−2^
	[77]	Ce‐doped BaTiO_3_	–	47–83 ms	–	76.1 mW cm^−^^2^
	[73]	ZnO/Cu_2_O	–	–	105	–
Organic semiconductor	[72]	DPP‐DTT	0.02697	–	10^4^–10^5^	–
	[12]	DNTT	–	0.6 s	10^6^–10^7^	0.5 mW cm^−^^2^
	[52]	P_13_	0.23	–	104	–
Nano‐material	[7]	CNTs	100 000	4.3–17.9 ms	–	24.4 W cm^−^^2^
	[10]	CNTs	≈12, ≈8	–	10^3^–10^4^	–
	[116]	CNT	–	–	>10^5^	–
	[70]	SWCNT	–	≈12 μs	>2.5 × 10^5^	2 mW cm^−2^
	[71]	CNT/Si_3_N_4_/Si	–	–	103	–
2D material	[11]	Graphene	–	56 ms–1.88 s	–	24.4 W cm^−^^2^
	[17]	Graphene	–	≈500 fs	–	–
	[107]	b‐AsP, MoTe_2_	≈145	600 ns–2.3 μs	–	–
	[108]	Various 2D materials	20–95	–	–	–
	[18]	MoTe_2_, h‐BN, Graphene	15.9	–	8 × 10^3^	–
	[19]	BP, MoTe_2_	–	<10 μs	>10^5^	–
	[20]	WSe_2_, h‐BN, Graphene	–	–	104	–
	[21]	MoS_2_, Ge	–	40–160 μs	≈10^5^	–
	[22]	MoTe_2_	–	1 kHz	–	–
	[24]	WSe_2_, h‐BN	–	20 ms	105	–
	[45]	MoS_2_, h‐BN, Graphene	–	–	1.5 × 10^7^	–
	[46]	WS_2_, MoS_2_, h‐BN	≈338	–	–	–
	[47]	BP, MoS_2_, h‐BN	≈147	15.7 ms–0.17 s	–	0.55 mW cm^−2^, 1.5 pJ
	[112]	MoS_2_ Monolayer	–	–	>10^5^	2.52 fJ
	[57]	PtSe_2_	1	–	–	–
	[58]	BP, SnS_0.5_Se_1.5_	–	15.7 ms–0.17 s	–	–
	[59]	FePS_3_	–	105–120 ms	–	0.18 mW cm^−^^2^
	[64]	MoS_2_	50	10 ns	10^5^	1 W cm^−2^
	[69]	Graphene	–	≈12 μs	–	2 mW cm^−2^
	[76]	MoSe_2_/Graphene	–	3.2–4.6 ms	–	1.77 mW cm^−^^2^
	[84]	MoS_2_	–	30–500 s	–	–
	[117]	MoS_2_/h‐BN	–	–	>10^5^	2.52 fJ
	[81]	PtS_2_/h‐BN/Graphene	–		>10^7^	
	[85]	α‐In_2_Se_3_	137.55	–	>10^6^	–
	[23]	Bi_2_O_2_Se	>20 000	–	–	1.1 mW cm^−2^
	[55]	Cr_2_Ge_2_Te_6_	–	1.3–2.2 s	–	–
	[115]	AgBiP_2_Se_6_	–	10 ms	–	–
Perovskite	[15]	Cs_2_AgBiBr_6_	–	–	103	130 μW cm^−2^
	[16]	MAPbI_3_, FAPbI_3_	–	40–680 μs	–	1 mW cm^−^^2^
	[103]	CH_3_NH_3_PbI_3_	–	0.26–0.48 s	1.2 × 10^3^	38.3 mW cm^−2^
	[27]	CH_3_NH_3_PbX_3_	–	<0.7 s	–	–
	[110]	Cs_4_PbBr_6_, PEDOT, Au	–	0.55–3.92 s	70	–
	[79]	CsPbBr_3_	–	–	>10^5^	–
	[83]	CsPbBr_3_/CsPbCl_2_Br	8.8	–	≈10^7^	–

### 2.1 In Organic–Inorganic Hybrid Perovskites

**Figure 2:**
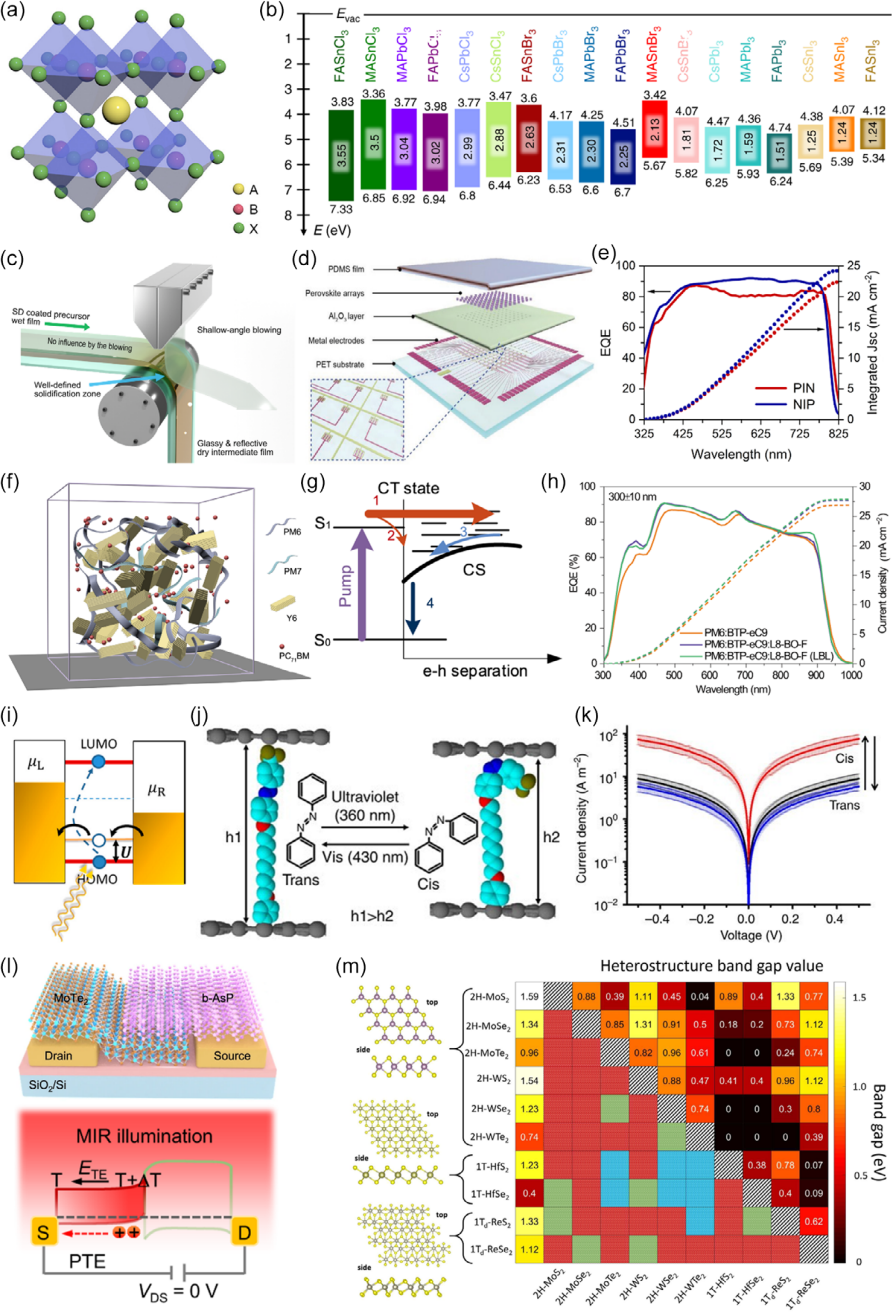
a) Perovskite structure. Reproduced with permission.^[^ ^100^ ^]^ Copyright 2022, Wiley‐VCH. b) Perovskite bandgap by composition. Reproduced with permission.^[^ ^101^ ^]^ Copyright 2019, Springer Nature. c) Roll‐to‐roll perovskite process. Reproduced with permission.^[^ ^102^ ^]^ Copyright 2024, Springer Nature. d) Solution process for perovskite. Reproduced with permission.^[^ ^103^ ^]^ Copyright 2019, Wiley‐VCH. e) EQE of PIN and NIP perovskite diodes. Reproduced with permission.^[^ ^104^ ^]^ Copyright 2023, Springer Nature. f) Molecule sketch in quaternary bulk heterojunction films. g) Solar cell electronic states and recombination processes. Reproduced with permission.^[^ ^105^ ^]^ Copyright 2021, Springer Nature. h) EQE spectra and integrated *J*_scs_ of bulk heterojunction device. Reproduced with permission.^[^ ^106^ ^]^ Copyright 2022, Springer Nature. i) Changes in the distance between two graphene electrodes (h1 and h2) when aryl azobenzene molecules are exposed to light. Reproduced with permission.^[^ ^34^ ^]^ Copyright 2018, American Chemical Society. j) Photoinduced current density–voltage graph (log scale) at low‐voltage range for the graphene‐aryl azobenzene device.^[^ ^42^ ^]^ k) The schematic of transport through a molecular junction shows that when exposed to light, the LUMO gets filled, causing the HOMO level to shift toward the Fermi level. Reproduced with permission.^[^ ^42^ ^]^ Copyright 2013, Springer Nature. l) Photoresponse of b‐AsP/MoTe_2_ heterostructure under MIR light at *V*_DS_ = 0 V. Reproduced with permission.^[^ ^107^ ^]^ Copyright 2023, Springer Nature. m) Bandgap values of 2D TMDC heterostructures from density functional theory–Perdew‐Burke‐Ernzerhof  calculations. Reproduced with permission.^[^ ^108^ ^]^ Copyright 2020, Springer Nature.

OIHPs have gained significant attention in the field of optoelectronics because of their remarkable characteristics, such as high absorption coefficients, adjustable bandgap, and cost‐effective solution‐based manufacturing.^[^ ^25^, ^26^ ^]^ Typically, OIHPs are structured as ABX_3_, where ‘A’ is a monovalent cation (e.g., CH_3_NH^3+^, Cs^+^), ‘B’ is a divalent metal cation (e.g., Pb^2+^, Sn^2+^), and ‘X’ is a halide anion (e.g., I^−^, Br^−^, Cl^−^) (**Figure** **2**a). The predominant OIHPs, known as MAPbI_3_, exhibits a bandgap of ≈1.6 electron volts (eV) and demonstrates exceptional quantum efficiency that is comparable to that of silicon.^[^ ^16^, ^27^ ^]^ The OIHP materials provide the advantage of easily adjusting the bandgap by modifying the composition or the ratio of its constituents (Figure 2b). Perovskites, unlike silicon and other inorganic materials, can be easily fabricated and manipulated in terms of their composition and physical absorption thickness using solution processing techniques (Figure 2c,d). The p–i–n junction is the most frequently employed structure in perovskite OELGs. This structure consists of electron and hole charge transport layers, which possess the property of being power free (Figure 2e).

OELGs uses light from ultraviolet to near‐infrared to distinguish between binary states 0 and 1 by setting specific photocurrent thresholds. Electron and hole movement, as well as output signal intensity, can be controlled by the material's bandgap and charge transport layers such as the electron transport layer and hole transport layer (HTL). Theoretically, all logic gate operations can be implemented by adjusting the device's structure and materials. However achieving stability comparable to silicon photodetectors is crucial for commercializing perovskite OELGs like CsPbBr_3_, Cs_2_AgBiBr_6_, and Cs_3_Bi_2_Br_9_/Cs_3_BiBr_6_.^[^ ^8^, ^15^, ^28^ ^]^ Although these OELGs have shown improved stability, retaining characteristics for hundreds of hours, further advancements are necessary.

### 2.2 In Conjugated Polymer Heterojunctions

Conjugated polymers with ionic–electronic conductive features are of paramount importance in soft electronics. Through the proper selection of device structures, channel materials, and electrolytes, organic semiconductors can serve as building blocks for a wide range of future logic gates, such as organic electrochemical transistors, electrochemical memory, and artificial synapses.^[^ ^29^, ^30^, ^31^ ^]^ For instance, Salleo's group first demonstrated a nonvolatile neuromorphic device with perfectly linear synaptic weight variance using PEDOT modified by PEI polymer.^[^ ^30^ ^]^ Zhang's group successfully realized a stable ambipolar polymer by tuning the ethylene glycol side chain length of the NDI‐2Tz polymer and demonstrated complementary inverters capable of operating at an extremely low voltage of 0.2 V.^[^ ^31^ ^]^ Similarly, polymeric semiconductors have contributed to the realization of OELGs. The active layer in organic solar cells and photodetectors is composed of a bulk heterojunction comprising conjugated polymers or dye donor materials and fullerene derivative acceptors (Figure 2f). Light absorption in these devices predominantly takes place via the donor, which stimulates electrons from the highest occupied molecular orbital (HOMO) to the lowest unoccupied molecular orbital (LUMO). The efficient separation of excitons, which is essential for the generation of electron–hole pairs, is accomplished by having a HOMO–LUMO energy offset that ranges from 0.1 to 1.4 eV (Figure 2g).

Conjugated polymers have an absorption coefficient that is similar to that of single‐crystal silicon (10^5^ cm^−1^). This similarity enables the creation of heterojunction devices using solution processing, similar to perovskites. These devices can be arranged in a vertical stack of photodetectors, allowing for flexible design of different photocurrent states and modes. Conjugated polymers are well‐suited for incorporating multifunctional OELG devices because they can effectively utilize a wide range of light wavelengths, spanning from visible to near‐infrared, as input for light (Figure 2h).

Conjugated polymers typically exhibit lower carrier mobility than inorganic photodetection materials, which poses challenges for achieving self‐powered operation. In order to achieve OELG operation, it is often necessary to use strong light pulses and apply additional electrical bias. Essentially, the extended carbon chains of conjugated polymers can be easily changed in shape by UV light, heat, and humidity. This can restrict the functioning of OELG photodetectors, especially when there is not a significant difference between the current in the absence of light and the current generated by light.

### 2.3 In Single‐Molecule Devices

OELGs based on SMDs, which utilize a molecular monolayer as a photoactive channel, have been highlighted as a novel solution to the miniaturization bottleneck of existing semiconductors.^[^ ^32^, ^33^ ^]^ Similar to conventional photodetectors, devices based on PCE^[^ ^34^, ^35^, ^36^ ^]^ and PVE are the most prevalent concepts for realizing OELGs using SMDs. Dubi's group empirically observed photoconductance in perylene tetracarboxylic diimide (PTCDI) molecules attached to Au electrodes (Figure 2i).^[^ ^34^ ^]^ Under illumination, electrons in the highest occupied molecular orbital (HOMO) level of PTCDI are excited to the lowest unoccupied molecular orbital (LUMO) level, leaving holes in the HOMO level. Conductance between the Au electrodes, initially small in the dark, dramatically increases due to these holes facilitating an electron penetration path between the electrodes. When an electrode of the SMD is selected in the form of a scanning tunneling microscope (STM) tip, high‐resolution molecular analysis is possible by tracking spatial photocurrent in real time. Kim's group placed a single free‐base phthalocyanine (FBPc) molecule on a NaCl thin film grown on an Ag substrate.^[^ ^35^ ^]^ They measured spatial photocurrent generation while scanning the tip under laser illumination, successfully visualizing an atomic‐scale resolution map representing the molecular orbitals of the FBPc molecule. Using the same approach, a diketopyrrolopyrrole molecule was analyzed with an STM Au tip electrode, demonstrating precise predictions of molecular orbital states that coincided with density functional theory calculations.^[^ ^36^ ^]^ To enhance photovoltaic conversion efficiency, donor–acceptor structures have been adopted to promote the photoinduced separation of electron–hole pairs.

Nichols's group pioneered the first optoelectronic SMD based on the PVE.^[^ ^37^ ^]^ This device features a heterojunction consisting of a low‐doped GaAs substrate and a 1,4‐phenylene(dimethanethiol) (1Ph1) single molecule, attached to an Au STM tip serving as a counter electrode. The device exhibits a high rectification ratio (>10^3^ at 1.5 V) and significant photocurrent generation under reverse bias, providing clear evidence for a PVE‐based SMD. Afterward, other single‐molecule/GaAs junctions or single‐molecule/Si junctions have been employed as key blocks for photovoltaic diodes.^[^ ^38^, ^39^ ^]^

Another promising SMD is designed based on a photoisomerization switching (PIS) mechanism. Diarylethene derivatives are well‐known photoswitchable chemicals that undergo 6π electron cyclization and decyclization under UV and visible light illumination, respectively.^[^ ^40^, ^41^ ^]^ It is noted that the electrical conductance of these chemicals highly depends on whether the ring is open (insulating) or closed (conductive). Based on these features, a novel photodetector can be realized by covalently binding the molecule to Au or carbon electrodes. Azobenzene derivatives are another candidate for PIS devices^[^ ^42^, ^43^ ^]^; their conductivity is switchable based on *cis*‐trans isomerization, with the former and latter forms induced by UV and visible light, respectively (Figure 2j). Due to the steric change caused by photon absorption, SMD‐based azobenzene demonstrates a dramatic resistance change depending on the light illumination conditions. Unlike PCE/PVE‐based devices, PIS devices maintain their conductance change even after the light stimulus is removed, exhibiting nonvolatile characteristics. Therefore, PIS devices would be highly beneficial for realizing in‐memory hardware by saving energy required to maintain the memory state (Figure 2k).

### 2.4 In Van der Waals Heterojunctions

vdW materials consist of strongly bonded, 2D layers weakly connected to form a 3D structure. A common vdW material, graphene, exhibits high carrier mobility and quantum confinement. Stacking different 2D materials leads to vdW heterojunctions with new electronic and optical properties (Figure 2i). Researchers have explored vdW heterojunctions using combinations of 2D materials such as graphene,^[^ ^44^ ^]^ hexagonal boron nitride (h‐BN),^[^ ^45^ ^]^ black phosphorus (BP),^[^ ^46^ ^]^ and transition metal dichalcogenides (TMDCs).^[^ ^47^ ^]^ These heterojunctions allow for bandgap tuning depending on the materials forming the junction (Figure 2j).

vdW materials demonstrate extremely accurate logic definition due to their very low dark currents and on/off ratios exceeding five orders of magnitude. Large‐area chemical vapor deposition and transfer processes are competitively priced compared to solution processing of organic materials. Since they can be deposited on silicon or oxide substrates, semiconductor processes allow for the downsizing and integration of devices, making vdW materials suitable for the fabrication of compact, low‐power OELG chips. Additionally, they inherently possess much higher mobility compared to bulk film optoelectronic devices, enhancing their applicability not only in optical computing but also in high‐frequency applications such as optical communications. However, the durability and sensitivity of their surfaces to gas and moisture in everyday environments pose constraints on OELG operation, necessitating further research into protective layers to mitigate these issues.

### 2.5 In Crystalline Silicon and Inorganic Materials

Crystalline silicon (c‐Si) is a foundational material in the semiconductor industry, particularly valued for its optoelectronic properties when implementing OELGs. It boasts a bandgap of 1.12 eV, enabling it to absorb a broad spectrum of light ranging from 250 to 1100 nm, which covers ultraviolet (UV), visible, and near‐infrared (NIR) light. This extensive range of light absorption is crucial for OELGs, as it allows for the use of binary or ternary optical inputs, making crystalline silicon a primary material in OELG research. Moreover, the integration of heterojunctions, which involve layering different materials with appropriate band offsets, can significantly enhance photocarrier separation and minimize recombination.^[^ ^48^ ^]^ This technique improves the absorption efficiency, particularly in the UV or NIR spectra, thereby boosting the spectral responsiveness of the devices.

Silicon p–n junctions have very few deep trap sites, thus ensuring consistent photocurrent generation and enhancing the reliability of OELGs based on the photovoltaic (PVE) mechanism. They also display high carrier mobility, which is essential for rapid charge transport and swift device response, crucial for the effective functioning of OELGs. c‐Si maintains stable performance under various environmental stressors such as heat, light, and humidity. One of the greatest advantages of using monocrystalline silicon in OELGs is that it facilitates of commercial‐scale integrated chip production, particularly useful when designing complex systems for OELGs. The scalability and integration capabilities of inorganic materials with existing semiconductor technologies facilitate the development of advanced integrated circuits that include OELGs, crucial for commercial applications.

## 3 OELG Operation by Resistance State Change Mechanisms

Photodetecting devices that absorb light and induce changes in the resistive state of a material exhibit multilevel implementations and electrical hysteresis making them highly suitable for various OELG‐in‐memory operations. These devices typically feature a metal–insulator–metal (MIM) structure, where a dielectric layer is sandwiched between two electrodes. They are often referred to as photonic synaptic or memory device due to their ability to use light in storing information. The electrical resistance of these devices primarily involves mechanisms such as conductive filaments (CF)^[^ ^49^ ^]^ and charge trapping.^[^ ^50^ ^]^ For CFs, resistance changes occur through the formation and dissolution of conductive pathways known as filaments. Newly formed filaments decrease the resistance, allowing electricity to flow easily. However, when these filaments are disrupted or destroyed, the resistance increases, inhibiting the current flow. Charge trapping occurs when charges, such as electrons or holes, are captured in specific regions of the material, affecting charge mobility and increasing resistance. The write operation of photonic logic‐in‐memory (LIM) based on charge trapping refers to the trapping and storage of photogenerated charges, representing a ‘1' or SET operation. The trapped and stored charges typically maintain their state for a considerable period of time. To revert this state back to a ‘0’ or RESET state, an erase operation is performed, causing the trapped charges to be released. Applying repetitive input pulses with insufficient values to reach the threshold for current/resistance/conductivity transformation induced by light enables the implementation of synaptic devices, multilevel resistance memory. Combining such memory with logic operations allows for the realization of photonic LIM properties. Another mechanism is phase‐change memory, which relies on the ability of certain materials to switch between amorphous and crystalline states. This switching is typically induced by temperature changes, which can be controlled by electrical pulses.

In this section, we explore how optoelectronic LIM operates by constructing resistance bits, focusing primarily on devices that utilize CF and charge trapping mechanisms.

### 3.1 By Formation of Conductive Filaments

CFs are widely used in data storage and switching devices such as resistive random access memory. There have been reports of research on OELGs that control this mechanism through light input. In these devices, materials absorb light to form CFs between two electrodes in the low resistance state (LRS) and can return to their original high resistance state (HRS, where the filament is disrupted) either when the light is blocked or through electrical stimulation. The process of filament formation induced by light and the subsequent recovery after light removal utilizes the characteristic resistance changes, enabling the achievement of bidirectional multilevel resistance states in devices with heterojunctions and charge transport interlayers. Materials such as TiO_2_, HfO_2_, and TaO_2_ have been extensively studied for CFs. Electrodes typically involve active metals like Ag and Cu (anode) and inert materials like Pt and ITO (cathode).

**Figure 3:**
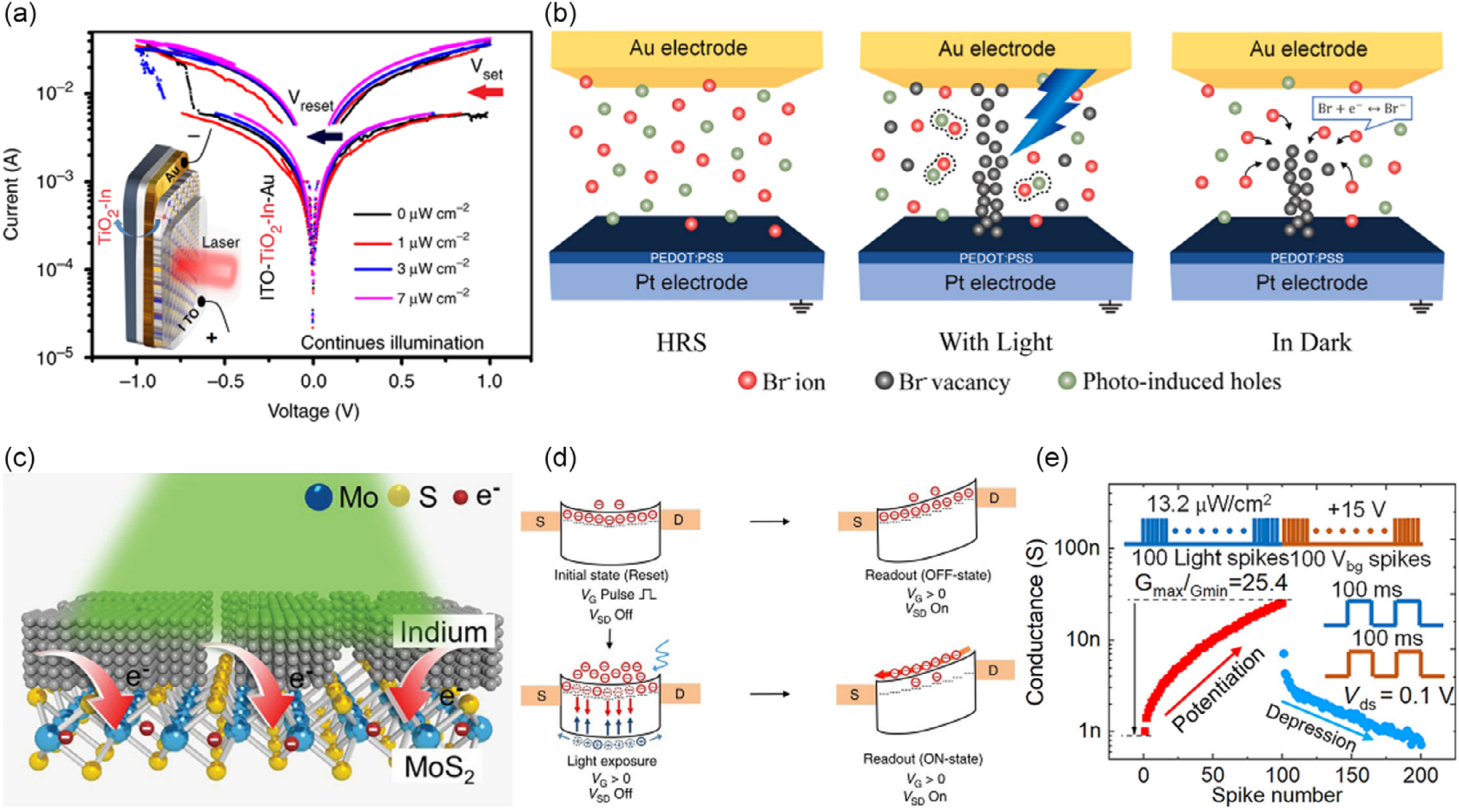
a) *I–V* curves of Pt/In‐doped TiO_2_/Au under 530 nm light with varying power densities. Reproduced with permission.^[^ ^109^ ^]^ Copyright 2019, Springer Nature. b) CF formation mechanism of Au/Cs_4_PbBr_6_/PEDOT:PSS/Pt optoelectronic device. Reproduced with permission.^[^ ^110^ ^]^ Copyright 2019, Elsevier. c) Surface charge doping in In/MoS_2_ device. Reproduced with permission.^[^ ^111^ ^]^ Copyright 2021, Wiley‐VCH. d) Energy band diagram of optoelectronic device operations (reset, light exposure, readout). Reproduced with permission.^[^ ^112^ ^]^ Copyright 2017, Springer Nature. e) Photonic potentiation and electric depression behaviors. Reproduced with permission.^[^ ^50^ ^]^ Copyright 2022, Wiley‐VCH.

A device that performs SET/RESET by forming CFs can be observed in the *I–V* curve, showing a memory window (**Figure** **3**a). The OELG is fabricated with an ITO/In‐doped TiO_2_/Au structure, where In^+^ intercalation is achieved through electrochemical doping using a solid electrolyte. Initially, the TiO_2_ thin film comprises a double‐layer stack of highly doped (top) and less doped (bottom) components, resulting in high electrical resistance between them. Under stimulation, the In^+^ cation migrates to the bottom TiO_2_ layer, increasing the conductivity of the layer and reducing the interfacial resistance between the two TiO_2_ layers. Consequently, the channel transitions to LRS (SET). When the applied voltage is reversed, the cation moves back and becomes reisolated in the top TiO_2_ layer, causing the channel resistance to revert to HRS (RESET process). Such operation is also observed in perovskites. Perovskite materials, when stimulated by light, can generate CFs through a process known as vacancy‐mediated hopping (Figure 3b), where halide anions (I^−^, Br^−^, and Cl^−^) migrate to fill vacancies. Electrical stimulation can switch these CFs from LRS to HRS. Notably, in 2D perovskites, the migration of halides involves a simpler diffusion path compared to bulk perovskites, where the process is more energy intensive and primarily occurs along the edges of PbX6 octahedra, enabling the implementation of low‐power OELGs.

### 3.2 By Charge Trapping

Charge‐trapping devices operate by capturing charge carriers like electrons or holes within defects or specific energy states in a material, influencing its electrical properties such as conductivity and resistivity.^[^ ^51^, ^52^ ^]^ This mechanism is crucial in nonvolatile flash memory and is sensitive to electric fields, temperature, and light, which can trigger the release of trapped charges. MIM or metal–insulator–semiconductor (MIS) structures encapsulate these charges within a dielectric layer. Applying a voltage induces an “off‐state” where charges are held at trapping sites, effectively storing binary data as “0" and “1.” Figure 3c illustrates a study showcasing the operation of a photonic synaptic device utilizing charge trapping, composed of an In/MoS_2_ structure. When light is applied externally, MoS_2_ generates electron–hole pairs, with the generated electrons being trapped in the multilayer MoS_2_ film and slowly released. By repetitively applying write/erase signals below the threshold, the amount of trapped charge and the amount of detrapped charge can be controlled.^[^ ^53^ ^]^ Using the photonic transistor based on MoS_2_/SiO_2_, they have successfully implemented a photonic synapse device capable of performing ON/OFF operations (Figure 3d). In the initial state (Reset) phase, a potential well is formed in the MoS_2_ channel, acting as a Schottky barrier due to its n‐type properties. When a gate pulse is applied, triggering the “Reset operation,” a considerable number of electrons in the channel are filled into the artificial trap sites due to the increase in the Fermi level. Upon the return of the gate voltage to 20 V, the electron concentration in the conduction band dramatically decreases due to the large number of trapped electrons (gate screening effect). During the readout for the off‐state (*V*_SD_ = 3 V) without illumination, the current level drops to below 4 pA. In the light exposure condition, when illuminated with a 450 nm laser pulse for 1 s, electron–hole pairs are generated in the MoS_2_ monolayer. Photogenerated holes can easily escape from the channel through an upward bending of the energy band, while trapped electrons are released through electron–hole recombination, allowing the accumulation of photogenerated electrons in the conduction band. Removing trapped electrons on the MoS_2_/SiO_2_ interface alleviates the gate screening effect, leading to the generation of more electrons in the conduction band and substantially more electrons being stored in the potential well with a longer lifetime. During the readout for the OFF‐state (*V*_SD_ = 3 V) without illumination, the current level drops to below 7.7 nA.

Figure 3e demonstrates the application of charge trapping characteristics in a photonic synaptic device using the pin/h‐BN/MoS_2_ vdW heterostructure. They induced potentiation by applying 100 pulses of light and implemented depression characteristics by applying 100 electrical field pulses. Consequently, for the implementation of logic‐in‐memory devices using photonic materials, superior mechanisms such as CF and charge trapping are advantageous for memory operations.

Organic semiconductors also have trapping sites influenced by defects, dipoles, and impurities, allowing control of LRS and HRS through light‐induced photogeneration and recombination.^[^ ^12^ ^]^ In principle, charge trapping devices may face issues with lifespan and operational reliability due to repeated operation cycles. Specifically, conductive polymers are susceptible to deterioration and are limited in their range of electrical and light usage due to inherently low mobility, which negatively impacts the speed and efficiency of OELGs. Consequently, OIHPs, which exhibit superior photoelectric conversion characteristics and enhanced durability, could serve as a viable alternative.

## 4 OELG Operation Through Combination of Various Mechanisms and Controls

**Figure 4:**
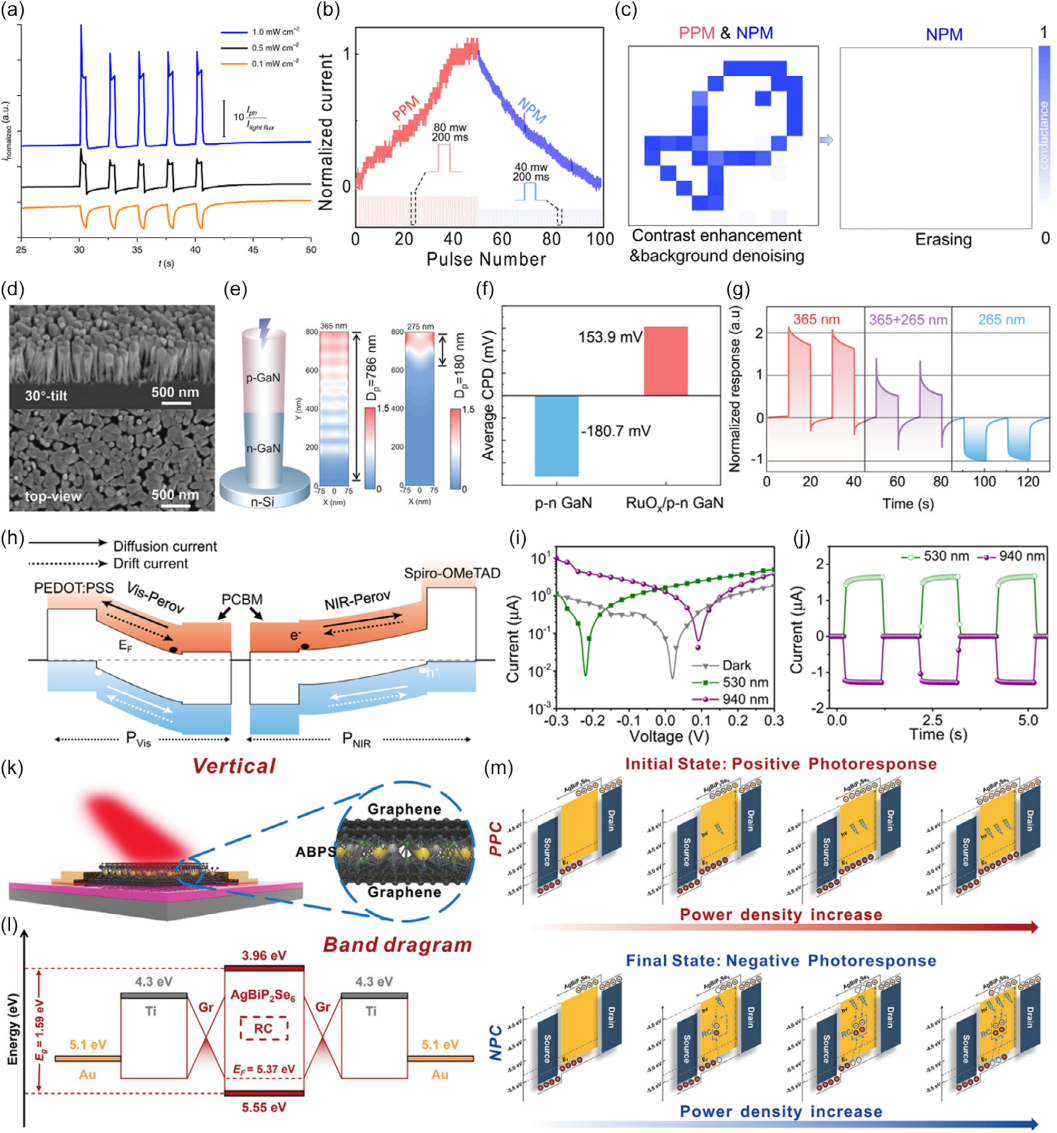
a) Light intensity‐induced photocurrent switching results. Reproduced with permission.^[^ ^113^ ^]^ Copyright 2020, Springer Nature. b) Optically induced potentiation and depression with different light pulses. c) Sensing, denoising, and enhancing a “fish” image in a 12 × 12 MSFP memory array, with erasable memory by light. Reproduced with permission.^[^ ^114^ ^]^ Copyright 2023, Springer Nature. d) SEM images of p–n GaN NWs on Si substrate. e) Penetration depths of 365 and 275 nm light and f) average CPD values in p–n GaN NW. g) Photoresponses of RuO_*x*_/p–n GaN photoelectrode under different illuminations. Reproduced with permission.^[^ ^28^ ^]^ Copyright 2023, Wiley‐VCH. h) Energy band diagram of p–*i*–n–n–*i*–p heterostructure perovskite device. i,j) *I–V* curves in the dark and under 530 and 940 nm. Reproduced with permission.^[^ ^16^ ^]^ Copyright 2022, Springer Nature. k) Schematic and l) band alignment of graphene/AgBiP_2_Se_6_/graphene photodetector. m) Mechanism of PPC and NPC response in the graphene/AgBiP_2_Se_6_/graphene photodetector with high‐drain–source voltage bias. Reproduced with permission.^[^ ^115^ ^]^ Copyright 2024, Wiley‐VCH.

Typically, in conventional photodetectors, photogenerated charges are extracted in a unipolar direction. This means that only one polarity, either positive or negative, is extracted when exposed to light. While this unipolar characteristic allows for distinguishing between 0 and 1, enabling AND and OR operations, it cannot implement complex operations such as NAND, NOR, XNOR, or XOR. Therefore, practically enabling multiple logic operations in a single device requires bipolar photoresponse. Bipolar photoresponse refers to the extraction of photoresponse in different directions depending on factors such as the intensity or wavelength of the input light or standby voltage (**Figure** **4**a). Implementing multifunctional logic through circuit designs that connect multiple photodetectors in series or parallel is possible. However, this approach is highly inefficient in terms of spatial and energy consumption.^[^ ^16^, ^28^, ^54^ ^]^ Bipolar photoresponse is crucial not only for the operation of multifunctional logic gates but also for the implementation such as memory or synaptic characteristics. For a device capable of bipolar photoresponse, as depicted in Figure 4b, write/erase operations can be performed with light of different intensities. However, for unipolar photoresponse components, auxiliary input signals such as writing with light and erasing with an electric field are required. OELG‐in‐memory devices can benefit from performing both write and erase operations with light, ensuring advantages such as parallel processing and operation speed. With advancements in this technology, the development of hardware components mimicking the human visual nervous system, as depicted in Figure 4c, becomes possible. Here, positive photoconductance (PPC) memory involves writing information by applying light from which positive‐direction photogenerated charges are extracted, while negative photoconductance memory involves erasing by applying light from which negative‐direction charges are extracted. Thus, extraction of bipolar photoresponse from a single component is a crucial factor in devices such as logic, memory, synaptic, and so on. Next, we will introduce various research demonstrations showcasing bipolar photoresponse.

Figure 4d shows the scanning electron microscope(SEM) image of the p–n junction structure of the RuO_*x*_/p–n GaN NW. Vertically grown RuO_*x*_/p–n GaN NWs exhibit bipolar photoresponse depending on the wavelength of incident light (Figure 4e). Under illumination at 365 nm, which penetrates almost the entire NW, charge carriers are generated in both the p‐GaN and n‐GaN segments. Electrons move toward the external circuit under the built‐in electric field, while holes predominantly drive oxidation reactions at the GaN/electrolyte interface, contributing to positive photocurrents. In contrast, under illumination at 275 nm, where light penetration is limited to the top p‐GaN segment, most carriers are generated near the surface of p‐GaN. Electrons dominate reduction reactions at the GaN/electrolyte interface, inducing negative photocurrents. Figure 4f analyzes the role of RuO_x_ in the bipolar photoresponse of RuO_*x*_/p–n GaN NWs, varying with wavelength. RuO_x_ loading lowers the surface Fermi level and facilitates electron transfer to the electrolyte. Suppression of electron trapping by surface states contributes to improved photoelectrochemical performance. As a result, RuO_*x*_/p–n GaN NWs induced bipolar photoresponse driven by the penetration depth of the incident wavelength, as illustrated in Figure 4g. Kim et al.^[^ ^16^ ^]^ created back‐to‐back diodes using perovskite materials, utilizing bipolar current characteristics that vary with different wavelengths (Figure 4h). The devices have a p^+^–i–n–n–i–p^+^ structure, which allows them to generate positive current under visible (VIS) light and negative current under near‐infrared (NIR) light. The p^+^–p–n–i–p^+^‐structured diode is an integrated device with a P_Vis_ diode and a *P*_NIR_ diode. It exhibited opposite photocurrent directions under the visible and near‐infrared illumination called bipolar photoresponse. When visible light is incident, electron/hole pairs are generated in the *P*_Vis_, and the electrons flow to the P_NIR_ side. In the case of near‐infrared light, electron–hole pairs are generated within *P*_NIR_ diode and it flows in the opposite direction in the case of visible light illumination due to the opposite built‐in potential, leading to bipolar photoresponse in the single device. This demonstrates the potential for using light to control polarity in photonic logic gate applications, benefiting from thin‐film formation and high integration. As shown in Figure 4h, when VIS light is applied, a negative polarity *V*_OC_ is produced, and with NIR light, a positive polarity *V*_OC_ is formed. At around 0.05 V, the currents for VIS and NIR are positive and negative, respectively. This bipolar current characteristic is effective within a readout voltage range of −0.2–0.1 V, as shown in Figure 4i,j. The aforementioned descriptions demonstrate the implementation of bipolar characteristics using device structures. Implementing bipolar characteristics through device structures has the advantages of relatively fast photoresponse and easy control. However, many studies are being reported that aim to reduce the number of vertical stacking layers.^[^ ^55^, ^56^, ^57^, ^58^, ^59^ ^]^ Figure 4k shows the implementation of bipolar response characteristics by applying a high‐drain–source voltage bias to the graphene/AgBiP_2_Se_6_/Graphene vdW vertical heterostructure. The energy band alignment of this device is shown in Figure 4l. In graphene/AgBiP_2_Se_6_/graphene vertical photodetector, the energy band alignment involves the formation of a smaller Schottky barrier height compared to parallel PPC photodetectors. This configuration enhances the overall optoelectronic performance by creating complete vdW contacts and an ultrashort channel length, which facilitates effective charge separation and transport. The heterostructure transitions from a positive photoconductive (PPC) response to negative photoconductive (NPC) response under a high‐drain–source voltage bias, attributed to the generation of trap states and subsequent quenching of photogenerated carriers. The operation mechanism is further detailed in Figure 4m. PPC photodetectors operate by absorbing photons when light is applied, generating electron–hole pairs that increase the conductance of the photodetector, resulting in a positive photocurrent. The increased concentration of free carriers in the device channel leads to higher photocurrent, which increases with the intensity of the incident light. Conversely, NPC photodetectors use a high‐drain–source voltage bias to induce trap states within the material. These trap states capture the photogenerated free carriers, reducing the number of carriers available for conduction. Consequently, the conductance decreases upon illumination, leading to a negative photocurrent that initially increases with light intensity but eventually saturates as the trap states become saturated.

The mechanisms for bipolar photoresponse focusing on device structure are compared. The RuO_*x*_/p–n GaN NW structure uses vertically grown NWs with p‐type and n‐type GaN segments, with RuO_*x*_ on the surface. 365 nm illumination generates positive photocurrent, while 275 nm creates negative photocurrent near the p‐GaN surface. RuO_*x*_ enhances electron mobility. The perovskite‐based back‐to‐back diode with p^+^–i–n–i–p^+^ structure produces positive current under visible light and negative under near infrared, enabling logic operations. The graphene/AgBiP_2_Se_6_/Graphene heterostructure achieves bipolar photoresponse with high‐drain–source voltage, enhancing charge separation. RuO_*x*_/p–n GaN and perovskite diodes are wavelength dependent, while graphene‐based structure varies with voltage. In summary, RuO_*x*_/p–n GaN NWs and perovskite‐based diodes exhibit wavelength‐dependent responses due to their layered and segmented structures, while the graphene‐based structure's response varies with voltage bias due to its vertical heterostructure. The perovskite diode's complex p^+^–i–n–i–p^+^ structure complicates fabrication, whereas the graphene structure is simpler but requires high voltage, impacting energy efficiency. The RuO_*x*_/p–n GaN NW structure, with its vertical growth and RuO_*x*_ surface layer, optimizes electron mobility and photoelectrochemical performance.

The materials and device structures that induce bipolar photoresponse are detailed in **Table** **3** and **4**. The characteristics and performance of these materials and device structures are compared. This will help to identify which materials and device structures give optimum performance under specific conditions.

**Table 3:** Overview of bipolar photoresponse studies induced by different materials and device structures. This table categorizes the types of modulators (input signals) that induce bipolar photoresponse.

References	Device structure	Mechanism	Magnitude
[28]	GaN p–n homojunction with RuO_*x*_	Wavelength selective	9.2 μA cm−^2^
[46]	BP/MoS_2_ heterostructure	Wavelength selective	1.2 A W^−1^
[113]	ITO/PET/CA–COH@ZnO	Light intensity induced	35.2 μA
[114]	Silk fibroin protein	Light intensity induced	320 mA cm^−2^
[115]	Graphene/AgBiP_2_Se_6_/Graphene	Voltage induced	489 704 A W^−1^
[54]	ZnO/Cu_2_O heterojunction	Voltage induced	6.5 × 10^−5^
[58]	BP/SnS_0.5_Se_1.5_	Voltage induced	5000

**Table 4:** Summary of the optoelectronic logic functions implemented by various materials and device structures.

References	Structural category	Device structure	Logic function
[4]	Nanostructure	Single CdS nanobelt	NOT, NOR, NAND
[6]		NW structure with CSi and PSi segments	AND, OR, NAND
[8]	Heterojunction	Using a ZnO and QDs active channel heterostructure	NOT, NOR, NAND
[13]		AZO/ZnO/PVK/PEDOT heterostructure on human hair	AND, OR, NAND
[19]		BP/MoTe_2_ vdW heterojunction	AND, OR, NOT
[21]		MoS_2_/Ge heterostructure JFET	AND, OR, multilogic calculations
[24]		WSe_2_/h‐BN/Al_2_O_3_ heterostructure phototransistor	NOR, XOR, NAND
[45]		Dual‐gate phototransistor based on WS_2_/MoS_2_ heterojunction	AND, OR, NOT
[78]		ITO/CdTe/SnSe/ITO heterojunction	OR, AND, NAND, NOR, NOT
[73]		ZnO/Cu_2_O heterostructure	OR gate
[76]		MoSe_2_/Graphene heterostructure	OR, AND
[74]	Thin‐film Device	MSM back‐to‐back Schottky contact device	AND, OR
[75]		Epitaxial ScN thin film	NOT, AND, OR, NAND, NOR
[16]		Back‐to‐back p^+^–*i*–n–p–p^+^ structure	AND, OR, NAND, NOR, NOT

## 5 Evolution and Innovations in Optoelectronic Logic Gate (OELG)

Logic gates are fundamental to modern digital computers, performing operations like AND, OR, and NOT. Despite their evolution, electronic logic gates face issues such as heat generation and signal interference, hindering speed improvements.^[^ ^60^, ^61^, ^62^, ^63^ ^]^ Optical computing, which uses light for logical operations, promises significant speed enhancements, ranging from several gigahertz to tens of terahertz, but faces challenges in compatibility and integration density.^[^ ^11^, ^64^, ^65^ ^]^

Efforts to improve computational speed using optoelectronic materials have been ongoing since the 1980s.^[^ ^66^, ^67^, ^68^ ^]^ Researchers have demonstrated various logic functions like AND, OR, and XOR using circuits composed of resistors, transistors, and lasers, respectively. Based on these studies, simplifying the circuits of OELGs and ensuring their versatility has become crucial. OELGs address these issues using light for signal input, making them suitable for neuromorphic hardware and parallel computing. They convert optical signals to electrical outputs and can perform a range of operations, including AND, OR, NAND, NOR, XOR, and XNOR. For example, an OR gate produces a threshold current when either input is on, while an AND gate generates significant current only when both inputs are on.

OELGs have evolved from demonstrating single functions in one device to efficiently implementing multiple functions within a single device. 1D‐1L OELGs perform various logic operations depending on structure, wavelength, and materials. The feasibility of optoelectronic logic has led to the development of MD‐ML OELGs, which perform operations like NOR and NAND within a single device. 1D‐ML OELGs advance efficiency by integrating three or more logic operations using various modulations such as electric fields, light, and heat, offering enhanced speed, cost efficiency, and overall performance.

This section discusses the evolution from initial single‐ and multiple‐photodiode logic gates to advanced architectures (MD‐ML, 1D‐ML) incorporating in‐memory functionality, offering insights into potential enhancements and research directions for the future of OELGs.

The logic operation functions from the cited studies are categorized and summarized in **Table** **5**. This table specifies the materials used and the device structures and also indicates whether bipolar photoresponse was achieved. The modulators controlling the bipolar photoresponse are listed, and an efficiency index is included to verify the presence of 1D‐ML. This efficiency index represents the number of logic operations driven by a single device, calculated by dividing the number of logic operations by the number of devices. Table 5 provides crucial data for a comprehensive comparison and analysis of the materials and structures used in various studies and their performance.

**Table 5:** Classification of logic operation functions from cited studies in this article. The table lists the materials used, the device structures, and indicates whether bipolar photoresponse was achieved. The modulators controlling the bipolar photoresponse are specified. For the verification of 1D‐ML, an efficiency index is included, which indicates the number of logic operations driven by a single device (number of logic operations/number of devices). The ‘–’ symbol indicates values that are ‘unspecified’ or ‘not mentioned’ in the references.

References	Logic function	Device Structure	Modulator	Polarity	Efficiency index	Applied Voltage	Device size
[4]	NOT, NOR, NAND	CdS nanobelt	Circuit	NO	1	0–15 V	2 μm × 10 μm
[6]	AND, OR, NAND	CSi and PSi segments	Circuit	NO	1	0–5 V	Diameter 25 nm, length 100 nm
[7]	Inverter, NOR	Silicon‐waveguide‐integrated CNT	Circuit	NO	1	–	length 100 μm
[8]	NOT, NOR, NAND	ITO/Al_2_O_3_/ZnO‐QDs/ITO	Circuit	NO	1	–	length 100 μm, width 1000 μm
[9]	OR	FTO/TiO_2_ NRs/CuInS_2_ NFs/Au	–	NO	1	–	–
[10]	Inverter, NAND, NOR	CNT/ITO‐TFT hybrid	Circuit	NO	1	–	length 170 μm, width 150 μm
[11]	AND, OR, NOT, NAND, NOR	CsPbCl_3_/graphene/Ge	Wavelength	YES	5	–	–
[12]	OR, AND	DNTT/DAE bilayer	Wavelength	NO	2	≈−60 V	–
[13]	AND, OR, NAND	AZO/ZnO/PVK/PEDOT	Circuit	NO	1	–5 V	length ≈5 μm, diameter ≈400 nm
[14]	AND, OR	p‐CsCu_2_I_3_/n‐Ca_2_Nb_3−*x*_Ta_*x*_O_10_	Circuit	NO	1	–	300 μm × 300 μm
[15]	OR	Ag/PMMA/Cs_2_AgBiBr_6_/ITO structure	–	NO	1	0–5 V	–
[16]	AND, OR, NAND, NOR, NOT	back‐to‐back p^+^‐i‐n‐p‐p^+^ structure	Wavelength	YES	5	0 V	200 × 200 μm^2^
[17]	AND, OR, NOR	hybrid graphene/D‐shaped fiber device	Circuit	NO	3	0–1 V	channel length 200 μm
[18]	AND, OR	MoTe_2_/h‐BN/Graphene SFG‐FET	Circuit	NO	1	−20 to 20 V	–
[22]	XOR	MoTe_2_/CuInP_2_S_6_/Au	Circuit	YES	1	≈1 V	≈11 μm
[23]	AND, OR, NAND, NOR	Bi_2_O_2_Se	Reference change	NO	1	–	22 μm
[24]	NOR, XOR, NAND	WSe_2_/h‐BN/Al_2_O_3_ heterostructure	Voltage	NO	3	–	length 15 μm
[110]	OR	Au/Cs_4_PbBr_6_/PEDOT/Pt	–	NO	1	−0.2 to 1.4 V	0.3 mm diameter
[64]	AND, OR, XOR, XNOR, NAND, NOR	Au/BP/Au with waveguide	Circuit	NO	1		≈10 μm
[77]	OR, AND, NOR, NAND	ITO/Ce‐BTO/Ag	Light	YES	4	–	–
[78]	OR, AND, NAND, NOR, NOT	ITO/CdTe/SnSe/ITO heterojunction	Light	YES	5	–	–
[73]	OR gate	ZnO/Cu_2_O heterostructure	–	NO	1	–	–
[69]	NOT, OR, AND	SWNT‐Si	Circuit	YES	1	–	–
[70]	AND	CNT/Si	–	NO	1	–	–
[76]	OR, AND	MoSe_2_/Graphene heterostructure	Material, circuit	YES	1	0.5 V	10 μm
[74]	AND, OR	GaN/Si	Circuit	NO	1	1 V	500 nm–5 μm
[75]	NOT, AND, OR, NAND, NOR	SNc/MgO, SNc:Mg/MgO	Circuit	NO	1	20 mV	–
[79]	AND, OR	CsPbBr_3_/MoS_2_/Si	Drain voltage	NO	2	0.01 V	–
[83]	AND, OR	Stepped FGs of perovskite QDs	–	NO	1	–	–
[84]	AND, OR	MoS_2_ transistor	Wavelength	NO	1	0 to −20 V	20 μm
[116]	AND	Au/CNT/Si	Voltage, Light	NO	1	–	–
[72]	NOR	Ag‐NWs/PDMS/DPP‐DTT/Au	–	NO	1	–	–
[117]	AND, OR, NAND, NOR	Au/MoS_2_/SiO_2_/h‐BN/Gr/Si	Voltage, Light intensity	NO	4	–	–
[118]	NOT, XOR, OR, ternary OR	Al_0.36_Ga_0.64_Na/Al_0.45_Ga_0.55_N/GaN/Si	Light intensity, Voltage, H_2_O_2_	YES	4	–	–
[85]	OR, AND	Pd/*α*‐In2Se_3_/SiO_2_/Si	Voltage	NO	2	±10 V	length 15 μm, width 2 μm
[86]	OR, AND	SiO_2_/MoS_2_/h‐BN/HfO_2_/Graphene	Voltage	NO	2	–	–

### 5.1 Single OELG for Single‐Logic Operation (1 Device to 1 Logic, 1D‐1L)

The first‐generation 1D‐1L utilized existing unipolar photoresponsive photodetectors and memory devices. Unipolar photoresponse characteristics can easily be implemented using materials such as Si, polymers, vdW materials, and perovskites as the photoconductive channel with Schottky or Ohmic contacts. Typically, these photodetectors generate current only in the presence of light and produce no current in its absence. The presence or absence of light alone determines the current flow, and the lack of mechanisms to adjust the amount or direction of the current restricts the performance of various logic operations. Most research has been focused on implementing OR gates, while the implementation of other gates such as AND has been more limited. An OR gate can detect current when at least one of two light sources is on, thus producing current under conditions where either or both inputs are ‘1’ (ON), and no current when both inputs are ‘0’ (OFF). This is the simplest form of implementation for an OR gate. Conversely, an AND gate requires both inputs to be ‘1’ for the output to be ‘1’. With unipolar materials, if any one of the inputs is ‘1’, current will flow, making it challenging to satisfy the AND gate condition where current should only flow when both inputs are simultaneously active. Although theoretically possible to design a device where current flows only when both light sources are activated, in practice, ensuring the precise simultaneity of inputs and preventing current induction by a single input present structural challenges.

Therefore, further research needs to progress toward designs incorporating multiple sensing materials and sophisticated device architectures to perform more complex logic operations. These developments will be discussed in subsequent sections, with Section 5.1 focusing on the fundamental materials, structures, and operational principles of key 1D‐1L OELG implementations.

**Figure 5:**
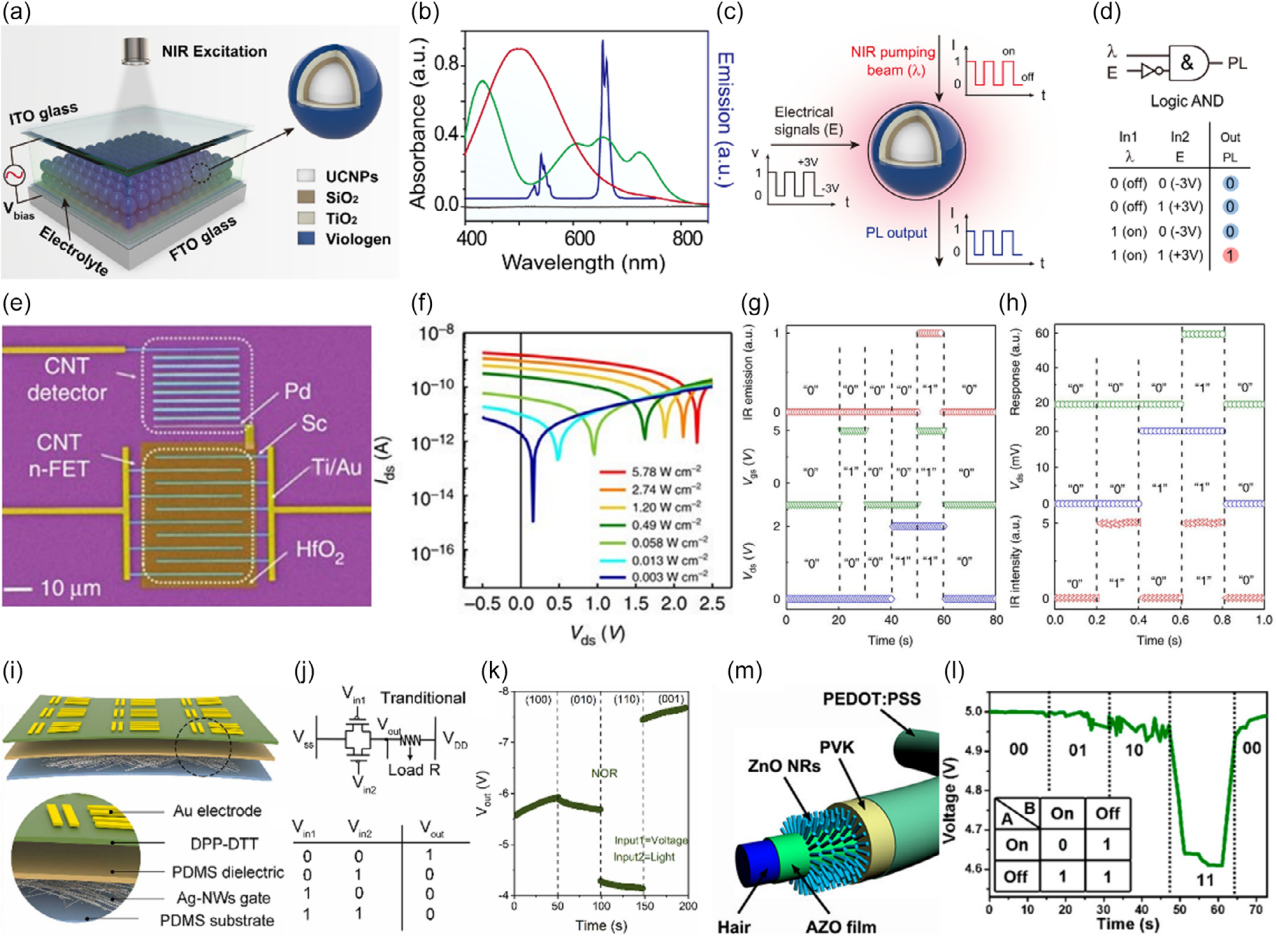
a) Electrochemical cell with viologen‐modified NaGdF_4_:Yb/Tm@NaYF_4_:Eu nanoparticles (Eu‐UCNPs). b) Emission spectrum of Eu‐UCNPs and absorption spectra. c,d) Logic AND gate operation using Eu‐UCNP/PV platform with NIR laser and electric field. Reproduced with permission.^[^ ^56^ ^]^ Copyright 2021, Springer Nature. e) False‐color SEM and f) photoresponse of a nine‐cell cascading photodetector with n‐FET. g) AND gate operation with *V*_ds_, *V*_gs_, and light output and IR light, h) *V*_ds_, and voltage output in CNT device. Reproduced with permission.^[^ ^116^ ^]^ Copyright 2017, Springer Nature. i) Schematic of DPP‐DTT OELG device. j) Conventional NOR logic gate circuit and truth table. k) NOR gate operation of DPP‐DTT device. Reproduced with permission.^[^ ^72^ ^]^ Copyright 2023, Elsevier. m) Schematic of AZO/ZnO device and l) NAND operation. Reproduced with permission.^[^ ^13^ ^]^ Copyright 2019, American Chemical Society.

A single device has limitations in independently controlling photocurrent generation from multiple photoresponsive layers, and very few studies of AND gate operation that require above‐threshold photocurrent output from only two optical inputs (`11` inputs) have been reported. Therefore, studies have been conducted that allow individual photoresponse control of binary inputs to implement AND gate operation in a single photodiode. Liu et al.^[^ ^56^ ^]^ implemented an optoelectronic AND gate using an electrochemical cell composed of NaGdF_4_:Yb/Tm@NaYF_4_:Eu nanoparticles (Eu‐UCNPs) and 1,1’‐bis(2‐phosphonoethyl)‐4,4’‐bipyridinium dichloride (PV) molecules (**Figure** **5**a). Eu‐UCNPs emit a dominant PL emission with peaks at 454 and 615 nm upon reception of 980 nm NIR light (Figure 5b). Meanwhile, the PV molecule can selectively absorb 615 nm light due to the electrochemical state change (PV^2+^ +anw^+^) only when a negative voltage is applied. By exploiting the reversible spectral resonance between these Eu‐UCNPs and PV molecules and setting the 980 nm NIR on/off and an alternating electric field of ±3 V as a binary input, the AND gate can be implemented to produce red emission at the 615 nm wavelength (output 1) only when the NIR is on, and the input is +3 V (Figure 5c,d).

To implement various gates including AND gates, researchers have studied junction devices using more than one light‐absorbing material from a single material, as introduced in Section 2. Particularly, carbon allotropes are very easy to shape from 1D NWs to 2D sheets and are straightforward to apply. This has led to the fabrication of junction photodevices such as Si waveguide‐integrated carbon nanotube (CNT) diode,^[^ ^7^ ^]^ Schottky diode,^[^ ^69^, ^70^ ^]^ and CNT/Si_3_N_4_/Si capacitors,^[^ ^71^ ^]^ which operate at high speeds. These devices have successfully implemented NOR, NOT, OR, XOR, adders, and digital‐to‐analog converters, applications that were impossible with single‐material photodetectors.

This study fabricated receivers and transmitters using CNTs to perform AND gate operations in two ways: light emission and absorption. The CNT‐based receiver uses s‐single‐walled CNTs (SWCNTs) as the optoelectronic material, Pd, Sc, Ti, and Au as metal electrodes, and HfO_2_ as the dielectric (Figure 5e). When illuminated with IR light (*λ* = 1,800 nm), the detector generates a photovoltage of 2.3 V and has a maximum responsivity of 0.67 A W^−1^. In contrast, the CNT transmitter, consisting of a controlling n‐type field‐effect transistor (n‐FET) and an emitter, regulates IR emission through electrical driving. The operational principle of the receiver involves the detector connected to the controlling n‐FET acting as an optical gate, generating tunable photovoltage based on the intensity of the input light (Figure 5f). The transmitter modulates emission intensity digitally via the controlling n‐FET. The receiver can be configured as an AND gate, with IR incident light and drain bias serving as inputs (Figure 5g,h). Results show that when both inputs are 1, the output current is 1 mA, representing logic 1. Similarly, the transmitter can also be configured as an AND gate with two electrical inputs, producing high IR emission when both inputs are 1. The CNT‐based 3D optoelectronic integrated circuit provides electrical isolation through a 20 nm HfO_2_ layer, enabling communication speeds exceeding 10 Gbps.

Recently, some studies have introduced flexible OELGs. In Figure 5i a light‐controlled NOR logic gate utilizing stretchable organic thin‐film transistors (OTFT) was developed. The DPP‐DTT‐based stretchable logic gate was fabricated by treating silicon wafers with octadecyltrimethoxysilane (OTS) and spin coating DPP‐DTT to form thin films. The bottom‐gate top‐contact stretchable OTFT was fabricated onto a PDMS substrate with Ag NWs as the gate electrode. While traditional NOR gates are implemented by connecting two transistors and a resistor in parallel, in this study, one transistor is removed using light as the input signal (Figure 5j). Voltage transfer curves were measured at various incident power densities using 808 nm NIR light as the input signal. When the light is on, the input signal is defined as “1,” and when it is off, it is defined as “0,” which, combined with the voltage input signal, performs the NOR gate function^[^ ^72^ ^]^ (Figure 5k). Additionally, in Figure 5m, a hierarchical heterostructure based on human hair was proposed. After depositing an AZO film on the hair, ZnO nanorods (NRs) were grown via a hydrothermal reaction to create an n‐type UV photoresponse layer. Subsequently, perovskite was deposited on the surface of the ZnO NRs to form a p–n junction, and PEDOT:PSS was used as HTL. Here, the AND and OR gates were implemented by connecting two heterostructures in series and parallel, respectively. In contrast, the NAND gate was implemented by connecting two heterostructures in series and using three electrodes, with a low output voltage observed only in the 11 state (Figure 5l). These studies have performed logic functions, such as AND, OR, NOR, and NAND. Most reported single OELG devices exhibit a predefined optical response (wavelength selectivity, optical current polarity, etc.) tailored to their structure, so they can only perform static logic gates. This limitation significantly reduces the spatial and energy efficiency compared to multifunctional 1D‐ML OELGs. When trying to apply the device in practical domains requiring various logic operation functions such as optical communications, multimodal light sensing, or optical computing applications, challenges in developing integrated high‐performance chips are posed.

Bu et al.^[^ ^73^ ^]^ developed a p–n diode with a CuInS_2_ (*p*‐type) and TiO_2_ (n‐type) heterojunction, responding to 365 and 475 nm light inputs. The diode functions as an OR gate, producing a photocurrent of 3.55 μA for 365 nm light, 1.10 μA for 475 nm light, and linearly adding these currents when both wavelengths are present. This dual‐band light response capability was also demonstrated with ZnO and Cu_2_O diodes, showcasing its potential for heterogeneous light source processors.

### 5.2 OELGs‐in‐Circuit for Multiple Logics (Multiple Devices‐Multiple Logics, MD‐ML)

The second‐generation OELG system was developed to address the limited logic operation capabilities of single devices and the difficulties in independently controlling the photoresponse to multiple light inputs. This system connects multiple OELGs in specific arrangements within a circuit, allowing each OELG to operate as an independent optical element based on the light input. This configuration simplifies the control of photocurrent using light or electric fields and provides the flexibility to arrange OELGs for multifunctional logic operations, surpassing the capabilities of a single OELG device. OELG‐in‐circuit systems can perform more diverse logic operations by adjusting the placement of phototransistors and photodiodes, making them a promising technology for the commercialization of OELG chips.

However, once the circuit is fabricated, the number of logic operations it can perform is fixed. This limitation is similar to existing electric logic gates, where the number of required circuits increases according to the type of logic operation. Additionally, the spot size of the light determines the density of logic gates per unit area on the OELG chip, posing challenges in implementing multiple logic operations in future downsized chips due to potential interference from binary inputs in a circuit composed of multiple photonic elements. Section 5.2 highlights the diversity and adaptability of OELG‐in‐circuit systems, providing detailed examples and clarifying the potential and challenges associated with these technologies.

**Figure 6:**
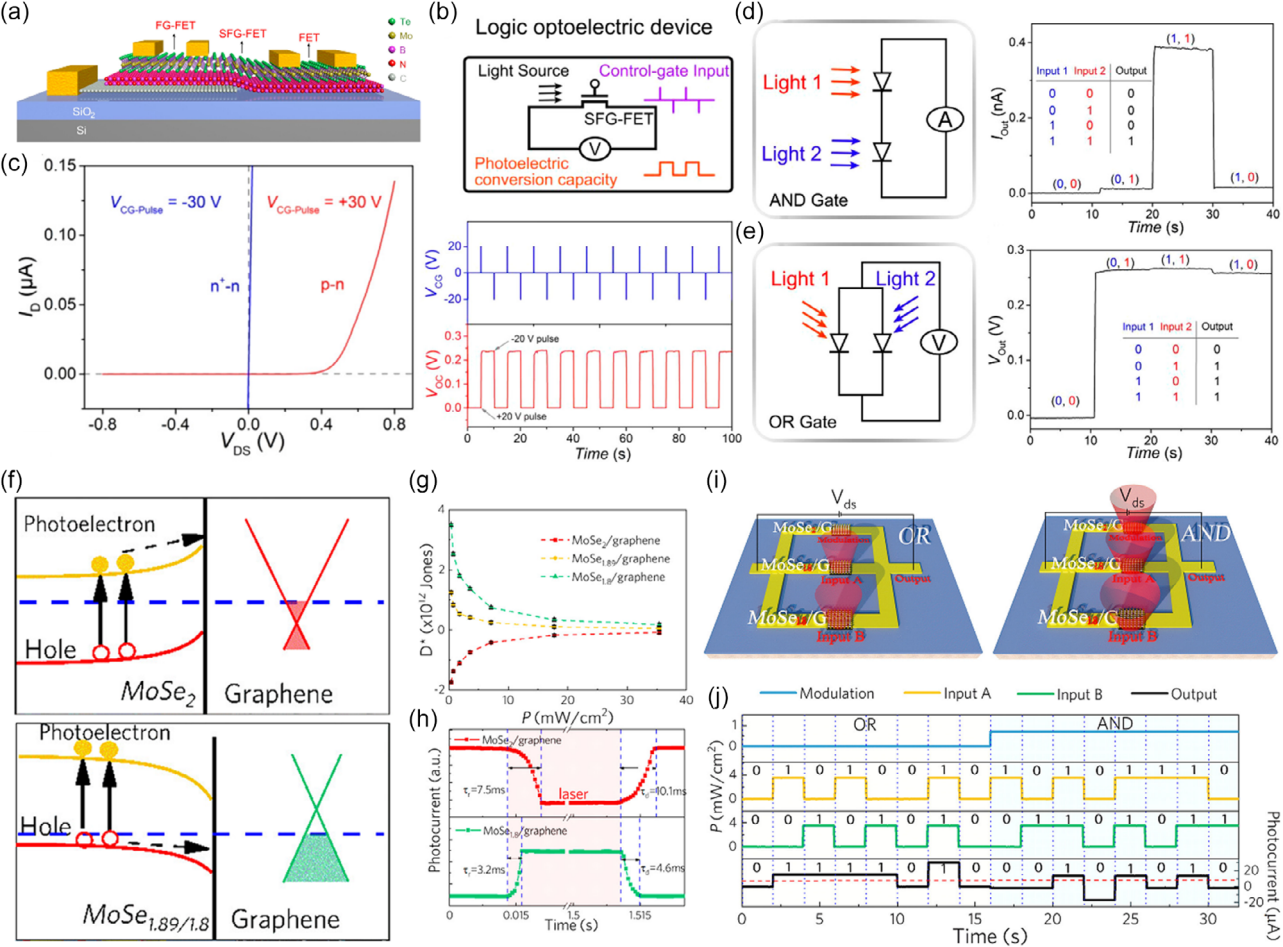
a) Schematic of FET, FG‐FET, and semi‐floating gate FET (SFG‐FET) architecture. b) Working mechanism of the logic optoelectric device and *V*_OC_ switching of SFG‐FET under continuous light (9.0 nW) with gate voltage pulses. c) Output curves of SFG‐FET after different control gate voltage pulses. d,e) AND/OR gate schematic with series‐/parallel‐connected p–n junctions and light inputs and measured output currents. Reproduced with permission.^[^ ^18^ ^]^ Copyright 2019, American Chemical Society. f) The band alignments of MoSe_2_/graphene and MoSe_1.89_/graphene. g) Specific detectivity (D*) of MoSe_2–*x*_/graphene with different ion fluences depending on the light intensity. h) Rise and decay time of photocurrent for MoSe_2_/graphene and MoSe_1.8_/graphene. i) Schematic diagrams of optoelectronic logic OR gate and AND gate (632.8 nm light). j) OR and AND logic operation in MoS_2−*x*_ device. Reproduced with permission.^[^ ^76^ ^]^ Copyright 2021, American Chemical Society.

Zhu et al.^[^ ^18^ ^]^ fabricated a semifloating gate (FG) FET by forming a partial graphene gate on a h‐BN/MoTe_2_ heterojunction using 2D sheet transfer technology (**Figure** **6**a). By applying a strong positive gate voltage of 30 V, hole trapping is induced only in the graphene gate region of MoTe_2_, converting the MoTe_2_ thin film from an n^+^–n junction to a p–n homojunction, resulting in a photodiode with an external quantum efficiency (EQE) of 0.5% at 0 V (Figure 6b,c). They implemented AND and OR gates by placing the converted p–n junctions in series and parallel^[^ ^74^, ^75^ ^]^ (Figure 6d,e).

Recently, systems that improve the functionality of OELG‐in‐circuit by actively controlling the photocurrent flow in the circuit by adding additional auxiliary circuits have attracted attention. Yanran Liu et al. fabricated a circuit with two MoSe_2_/graphene transistors (A1, A2) and one MoSe_1.8_/graphene transistor (M) in a three‐row parallel structure (Figure 6f).^[^ ^76^ ^]^ MoSe_2–*x*_ can convert most of the carriers transferred at the MoSe_2–*x*_/graphene interface from electrons to holes depending on the compositional change of Se (0 < *x* < 0.2), so that A1, A2 devices (p‐type), and M devices (n‐type) have opposite directions of photocurrent (Figure 6g,h). Since A1 and A2 devices are composed of parallel circuits, they perform an OR gate for each binary input. On the other hand, when light is incident on the M device, the photocurrent generated in the opposite direction of the A1 and A2 devices cancels out the photocurrent of the entire circuit, the photocurrent exceeding the threshold is generated only when light is finally incident on both A1 and A2, and AND gate operation is possible (Figure 6j).

He et al.^[^ ^64^ ^]^ integrated silicon waveguides with 2D material, BP, to realize MD‐ML logic operations. The operation of the logic gate relies primarily on two principles. First, when light is irradiated onto the waveguide, BP generates a light trigger, activating the photocharge transfer between the waveguide and BP. Second, this charge transfer is electrically controlled, and the voltage is adjusted based on the light input applied to the waveguide. Thus, the combination of BP and waveguides enables various logic operations such as AND, OR, XOR, etc., under different input conditions. This offers advantages of fast response times of 230 MHz and small device sizes of 1.55 μm, highlighting the significance of integrating optoelectronic materials with silicon waveguides for greatly enhancing response times.

Kim et al.^[^ ^6^ ^]^ achieved an OELG‐in‐circuit using single‐crystal silicon NWs. They employed a metal‐assisted chemical etching method to selectively form an amorphous silicon (a‐Si) channel at the center of a single‐crystal Si NW, resulting in a high‐sensitivity photodetector with an on/off ratio exceeding 10^6^. When two a‐Si channels in a single Si NW are connected in series, the high electrical resistance prevents a single light input from generating a photocurrent above the threshold, producing an output of 0 for 10 and 01 inputs. However, when light is applied simultaneously, the resistance decreases, generating a photocurrent above the threshold and implementing an AND gate with an output of 1 for the 11 input. Conversely, connecting the channels in parallel allows a single optical input to generate a photocurrent above the threshold, thus implementing an OR gate. Additional circuits demonstrated NAND and NOT gates by placing two or more devices in specific configurations.

### 5.3 Reconfigurable OELGs (one Device, Multiple Logics, 1D‐ML)

Reconfigurable logic systems, such as field‐programmable gate arrays, allow the internal interconnections of basic logic blocks to be reversibly programmed and the operations were actively modified even after fabrication. In semiconductor design, these systems have limitations such as high power consumption and low device density. However, optical‐based logic operation systems in which the density of the system is determined by the spot size of the light, such as OELG or OLGs, can solve the limitations such as low chip density and low versatility of static OELGs. Therefore, they are evaluated as third‐generation OELG‐driven systems. The reconfigurable OELG system does not have fixed logic operation according to the device structure and circuit arrangement and can actively convert the logic operation using additional stimuli such as electric fields and light.^[^ ^77^, ^78^ ^]^

**Figure 7:**
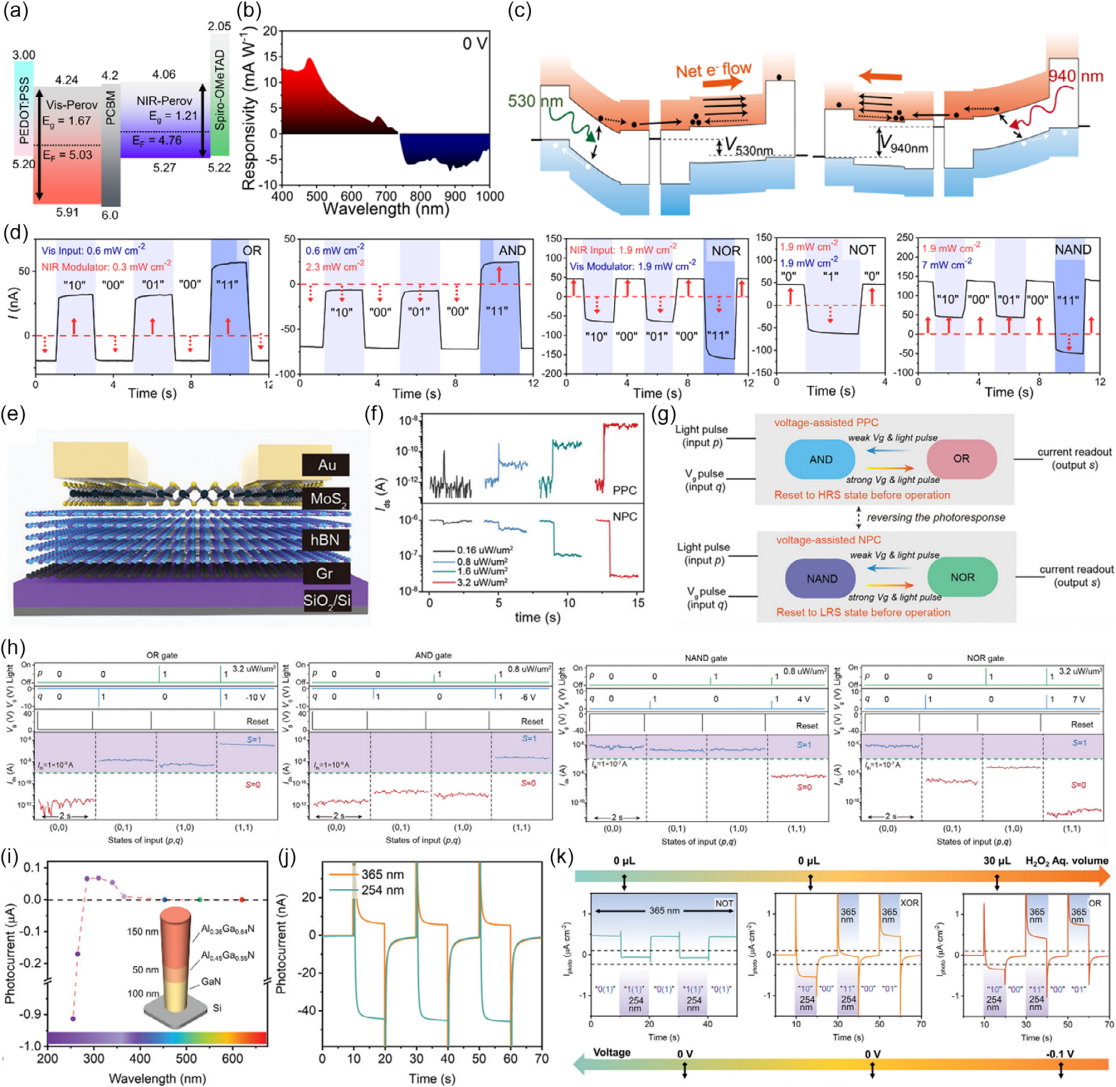
a) Energy band diagram of perovskite p^+^–*i*–n–p–p^+^ structure and b) bipolar spectral photoresponse from 400 to 1000 nm. c) Energy band diagram of heterostructure perovskite diodes and d) logic operations: OR, AND, NOR, NOT, NAND.^[^ ^16^ ^]^ Copyright 2022, Springer Nature. e) Schematic of MoS_2_/h‐BN/Gr/SiO_2_/Si FG transistor. f) NPC and PPC characteristics at different light pulse powers in MoS_2_/h‐BN transistor and g) logic gate operation schematic. h) Logic operation of MoS_2_/h‐BN transistor OR‐AND and NAND‐NOR. Reproduced with permission.^[^ ^117^ ^]^ Copyright 2022, Wiley‐VCH. i) Current signals of p‐AlGaN/n‐GaN NWs under various LED wavelengths and NW. j) Bipolar photoresponse with 254 and 365 nm LED in NW device and k) logic operations: NOT, XOR, OR depending on the voltage and H_2_O_2_. Reproduced with permission.^[^ ^118^ ^]^ Copyright 2023, Wiley‐VCH.

For the first time, Kim et al.^[^ ^16^ ^]^ developed a reconfigurable OELG that can perform multiple logic operations only in a single device. They fabricated a p^+^–p–n–*i*–p^+^‐based back‐to‐back diode by vertically stacking a visible light‐responsive n–*i*–p^+^ photodiode in the form of PCBM/MAPbI_3_ (Vis‐Perov)/PEDOT and a p^+^–p–n device and Spiro‐OMeTAD/FA_0.5_MA_0.5_Pb_0.4_Sn_0.6_I_3_ (NIR‐Perov)/PCBM (**Figure** **7**a). It has the bipolar photoresponse property with opposite photocurrent directions in the visible and near infrared. (Figure 7b). When visible light is incident, electron/hole pairs are generated in the Vis‐Perov containing n–*i*–p^+^, the photogenerated electrons flow through the n‐type PCBM to the opposite diode, and the holes flow through the p‐type PEDOT to the electrode, generating a sizeable photocurrent (Figure 7c). In the case of near‐infrared light, it passes through the Vis‐Perov layer and reaches the NIR‐Perov, where the built‐in potential in the p^+^–p–n device causes the electrons to flow to the opposite diode and the holes to flow to the Spiro‐OMeTAD layer, each in the opposite direction of the incident visible light. Using this bipolar photoresponse, the team introduced a breakthrough factor called an “optical gate modulator” (OGM), in which the photocurrent from the OGM input and the reverse photocurrent from visible or NIR light can cancel out the output, resulting in multiple bit states. The degree of the output offset is proportional to the intensity of the OGM wavelength, and using this principle, they proposed a system that can realize different logics only in a single device. If the on/off of two visible lights is a binary input, and the intensity of the NIR OGM is weak, the degree of offset is weak, and the perovskite OELG can perform OR logic with a positive photocurrent flowing from a single optical input. Conversely, if the intensity of the NIR‐OGM is higher, there is enough negative photocurrent to cancel out the positive photocurrent, and the positive photocurrent can only be observed when both visible lights are turned on simultaneously. The threshold current of 0 A means that the NIR OGM causes the device to switch to an AND gate. Using this principle, five logic gates (AND, OR, NAND, NOR, and NOT) were successfully demonstrated for the first time in a single device (Figure 7d). However, back‐to‐back diode‐based OELG has limitations such as relatively slow switching speed and balancing the equivalent photoresponse under two different light wavelengths, restricting achieving enough operational margin in light irradiance. In addition, such a device structure can suffer from severe resistance increase and interfacial charge or ion build‐up due to the multi‐interface layered structure which has multi‐interfacial properties.

The MoS_2_/h‐BN/Gr vdW heterostructure FG transistor utilizes MoS_2_ as the channel, h‐BN as the tunnel barrier, monolayer graphene as the FG, SiO_2_ as the gate dielectric, and Si as the control gate (Figure 7e). This device enables multilevel ion trapping and analog‐like conductivity switching with low‐voltage signals. Investigation of its optical response using 532 nm light revealed phototriggered positive and negative photoconductivity, suggesting the device's potential for reconfigurable Boolean logic operations using optical signals (Figure 7f,g). Based on this, a photonic logic‐in‐sensor device capable of performing reconfigurable Boolean logic functions, including “AND–OR” and “NAND–NOR” logic gates, was proposed and successfully demonstrated (Figure 7h). Moreover, the device's logic operation results vary with the intensity of external stimuli, and various logic gates can be reconstructed by modulating the voltage pulse method. vdW heterostructure OELG has advantages in fast operation speed under optical and electrical stimuli; however, with respect to commercialization, the operation voltage (4–10 V) is still much higher than electronic logic gates and the wavelength and irradiance for generating enough photoresponse is still limited to the short visible light region and high power. Moreover, the vdW structure is unfavorable to fabricate highly integrated circuits in a large area due to its main fabrication method such as pattern or thin‐film transfer.

The photodetector based on p‐AlGaN/n‐GaN NWs enables a reprogrammable OLG (Figure 7i). It allows for the realization of various logic operation modes by adjusting various inputs such as light intensity, applied voltage, electrolyte conditions, and electrode (co‐catalyst) (Figure 7j). This OLG implements binary “NOT,” “XOR,” and “OR” logic gate modes based on different programming inputs (Figure 7k). When light with wavelengths of 254 and 365 nm is input via light‐emitting diodes (LEDs), the logic state of the output is determined. A ternary input and ternary output “OR” gate is also demonstrated by adjusting a constant bias voltage. The third stimuli is electrolyte condition. As the concentration of H_2_O_2_ increases, GaN separates H_2_O_2_ into water and oxygen through a photocatalytic reaction and then more holes are generated under 365 nm irradiation. As a result, the level of photocurrent at the 365 nm wavelength increases, and the logic function switched from XOR to OR. Furthermore, this reprogrammable OLG optimizes power consumption to maintain high operating speeds, exhibiting excellent performance in data processing. NW‐based logic gates produce excellent bipolar photoresponses through bandgap separation by alloying within a single‐material matrix, but the usable wavelength range is limited to the UV region, and the device design for fabricating integrated circuits is required in practical aspect.

Cheng et al.^[^ ^79^ ^]^ developed a reconfigurable OELG system using gate voltage, drain voltage, and light illumination as stimulus pulses. The system features a CsPbBr_3_/MoS_2_ heterojunction channel with an ionic gel‐based electrolyte as a dielectric gate, exhibiting synaptic properties and generating different EPSCs based on stimulus timing. With gate voltage and 405 nm light as binary inputs, an AND gate is achieved at drain voltages below 0.1 V, and an OR gate at 0.2 V or higher. This synaptic transistor‐based OELG enables reversible reconfiguration of AND, OR, and XNOR logic operations.

### 5.4 Nonvolatile OELGs

Typical OELGs have the advantages of high speed and low energy consumption, but it is difficult for them to overcome the limitations of existing computer systems such as communication delay between central processing unit (CPU) and memory. To address this issue, the researchers developed integration of logic function and memory to eliminate the physical distance between the CPU and memory. In this respect, multibit memory (MBM) and synaptic potentiation/depression property are recently highlighted. Although the two aforementioned methods are researched to implement analog computing, researchers aimed to develop a new system that integrates logic functions and memory based on the multibit and synaptic properties. Consequently, LIM devices and nonvolatile reconfigurable OELGs (n‐vr OELGs) are widely studied.

LIM devices integrate logical operations and memory functions within a single structure, allowing for simultaneous data processing and storage. These devices typically utilize FGs or charge trapping mechanisms for nonvolatile storage. The FGs and trapping layers are formed by stacking or mixing multiple materials, making them suitable for implementing multibit states. This feature is particularly advantageous for integrated applications requiring high data density and efficiency, such as in‐memory computing or edge devices. To maximize efficiency in terms of size and power consumption, future LIM devices should be developed as integrated chips. Additionally, research should focus on hybrid materials and structures that can maintain long‐term data stability, even under high‐temperature conditions.

On the other hand, n‐vr OELGs are designed to dynamically reconfigure logical functions through the simultaneous application of light or a combination of light and electric field stimulation. While these devices also use charge trapping mechanisms for data storage, they place a greater emphasis on reconfigurable logical functions. This makes them ideal for adaptive logic processing in real‐world computing systems. It is crucial that the dynamic reconstruction of logic functions remains consistent in subsequent operations. For future advancements, improving the precision of logical reconstruction, enhancing the retention time and speed of state reconstruction, and minimizing power consumption will be key challenges.

#### 5.4.1 Logic‐in‐Memory Devices

Before LIM devices were studied in earnest, MBM devices, which can realize multibit resistance states using nonvolatile features and light pulses, were studied.^[^ ^47^, ^80^, ^81^ ^]^ Tran et al.^[^ ^44^ ^]^ fabricated MBM devices with more than 18 states using MoS_2_ transistors with graphene as FGs and succeeded in realizing stable devices with retention time of more than 3.6sto^4^ s and program‐erase cycles of more than 10^4^ using h‐BN. The advent of LIM devices marked a significant advancement, as these devices integrated logic operations with memory functions.

**Figure 8:**
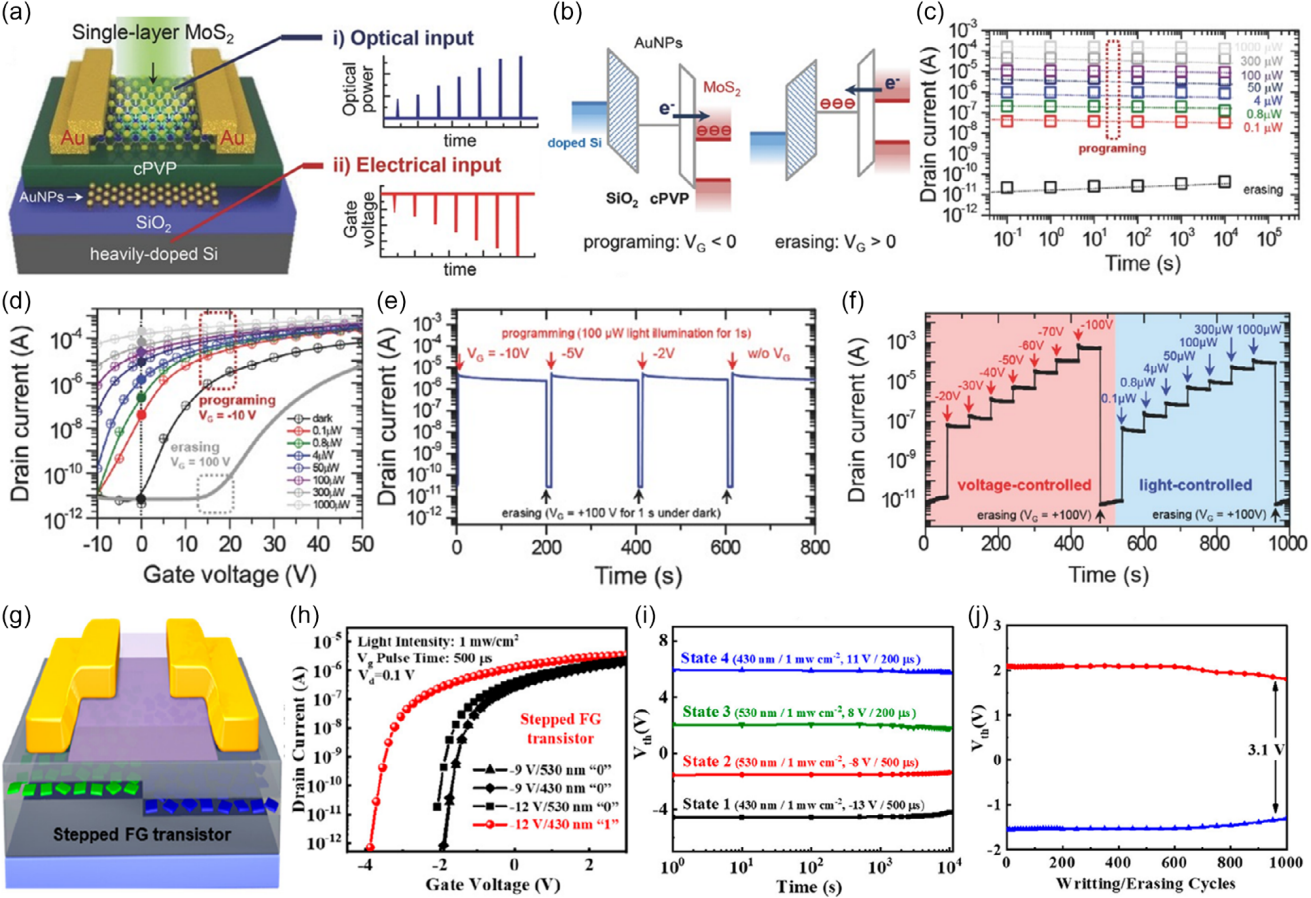
Schematic of MoS_2_ a) device structure and b) band diagram controlled by voltage. c) Retention time and d) transfer curve of MoS_2_ optoelectronic memory device. e,f) Light‐ and voltage‐controlled retention. Reproduced with permission.^[^ ^82^ ^]^ Copyright 2016, Wiley‐VCH. g) Schematic of stepped FG transistor and h) transfer curve. i) Retention characteristics of multiple memory states and j) photoelectric writing/erasing endurance performance of the stepped FG transistor. Reproduced with permission.^[^ ^83^ ^]^ Copyright 2022, American Chemical Society.

Lee et al. realized a multibit system with an Au nanoparticle (Au NP) floating‐gated MoS_2_ transistor whose resistance value can be linearly modulated by stepwise intensity changes of light and voltage (**Figure** **8**a).^[^ ^82^ ^]^ When light is applied to MoS_2_, an electron–hole pair is generated, and electrons and holes are separated by a crosslinked poly(4‐vinylphenol) (cPVP) dielectric layer toward the MoS_2_ and Au NP FGs, respectively (Figure 8b). At this point, only holes are selectively recombined with the remaining electrons in the Au NP FG, ultimately lowering the resistance of the MoS_2_ channel (programming). This programming state is erased by a bias pulse of 100 V or more, making it a memory device that can store data with light (Figure 8c–e). In this case, the programmed state (drain current) is semipermanent, and at the same time it is characterized by a linear increase with the amount of light, which succeeds in implementing a nonvolatile multibit (Figure 8f).

Pei et al. developed a stepped FG transistor, which implements LIM function in which logic operations are performed, and the results are stored at the same time (Figure 8g).^[^ ^83^ ^]^ The stepped FG transistor consists of two different FGs inserted into one device, one being an IGZO channel/7 nm Al_2_O_3_ dielectric/CsPbBr_3_ QD FG and the other being an IGZO channel/10 nm Al_2_O_3_ dielectric/CsPbCl_2_Br QD FG. Since the bandgap of the two FGs is different (CsPbBr_3_ QD: 2.3 eV, CsPbCl_2_Br QD: 2.8 eV) and the thickness of the dielectric layer is different, the former can be programmed with a combination of a low negative bias (<9.5 V) and a long‐wavelength pulse (>430 nm), while the latter can induce the desired electron transfer by simultaneously applying a bias of 9.5 V or more and a light pulse with a wavelength shorter than 430 nm (Figure 8h). Using this setup, the researchers implemented an AND gate where the gate voltage magnitude (0 and 1 for >−9.5 V and <−9.5 V, respectively) and the incident light wavelength (0 and 1 for >430 and <430 nm, respectively) serve as binary inputs, with the threshold voltage (*V*_th_) of the transistor as the output (Figure 8i). This LIM‐based OLEG system demonstrates maintaining a memory window even after 1000 cycles of writing and erasing. The device retains a significant memory window after numerous cycles, highlighting its potential for reliable and durable memory applications in advanced optoelectronic systems (Figure 8j).

#### 5.4.2 Non‐Volatile Reconfigurable OELGs (n‐vr OELGs)

N‐vr OELGs are systems that enable the conversion of logic operations and the permanent maintenance of these converted logic states using charge trapping mechanisms. These devices can perform multiple logic operations within a single device or circuit. The primary advantage of n‐vr OELGs is that an initial stimulus determines their computational function, which can then be maintained without additional power.

**Figure 9:**
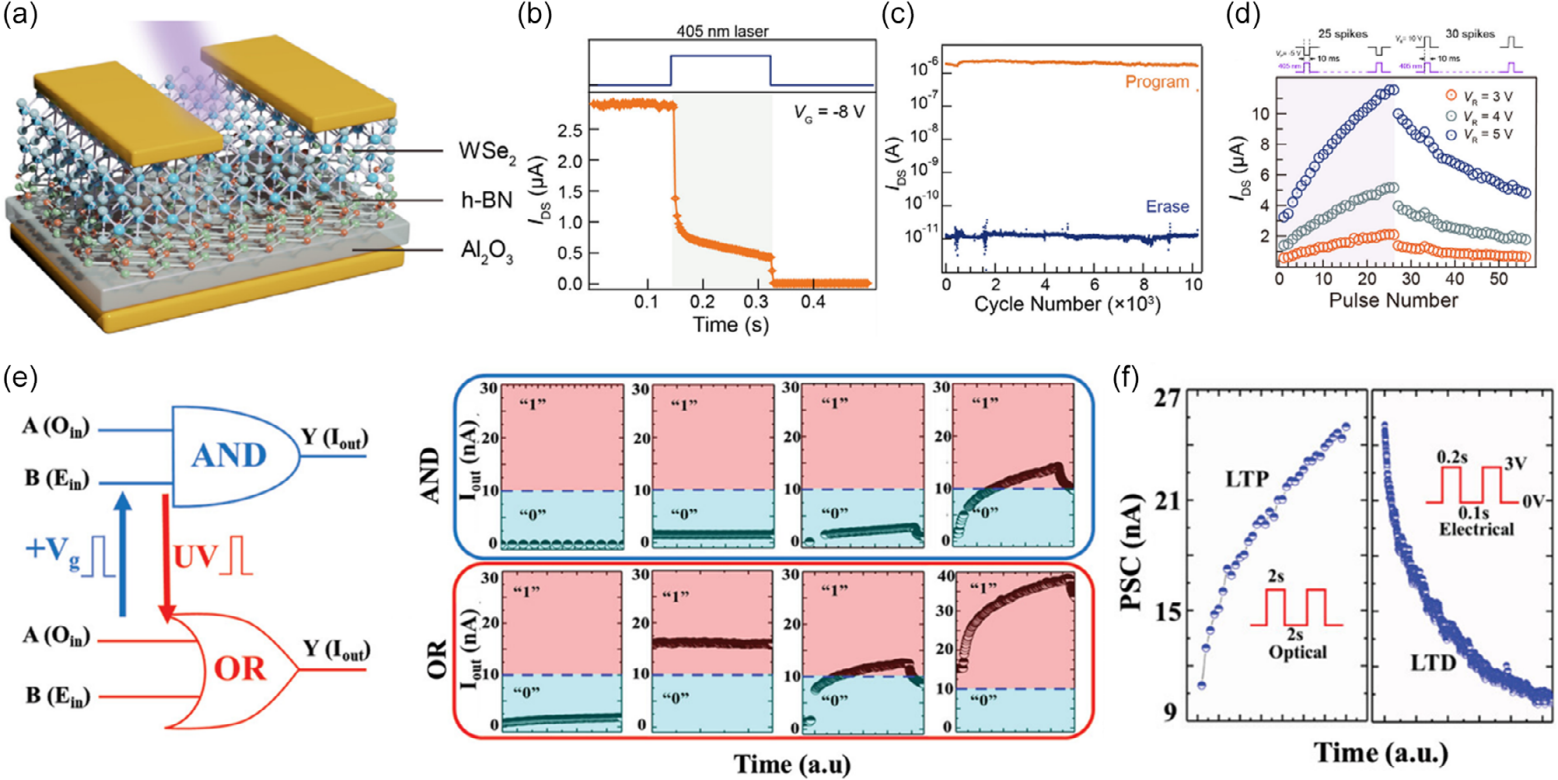
a) Schematic of the WSe_2_/h‐BN phototransistor and b) *I*_DS_–*t* curve under *V*_G_−8 V with a 405 nm laser pulse. c) Endurance test and d) potentiation and depression curve under *V*_R_ using spike sequences. Reproduced with permission.^[^ ^24^ ^]^ Copyright 2022, American Chemical Society. e) AND and OR gates are implemented using the mechanism where the extraction of photogenerated carriers under UV light varies based on the applied gate voltage (*V*_g_). f) LTP: Increase in PSC with 35 optical pulses and LTD: Decrease in PSC with electrical gate pulses. Reproduced with permission.^[^ ^84^ ^]^ Copyright 2023, Wiley‐VCH.

Miyata et al.^[^ ^25^ ^]^ presented an n‐vr OELG using WSe_2_/h‐BN/Al_2_O_3_ heterostructure phototransistors (**Figure** **9**a). This n‐vr OELG can nonvolatilely control the ambipolar characteristics in the presence or absence of light. Photogenerated holes in WSe_2_ are semipermanently trapped at the h‐BN/Al_2_O_3_ interface, creating an electrostatic modulation effect that significantly increases the conductance of the WSe_2_ channel compared to that in the absence of light (Figure 9b). Based on this principle, the research team realized a reconfigurable multilogic gate that can perform NAND, XOR, and NOR operations by changing the read voltage applied to the gate. Further, they developed a secure optoelectronic circuit that uses gate voltage modulation as an encryption key. The nonvolatile phototransistor utilizes a WSe_2_/h‐BN/Al_2_O_3_ vdW heterostructure to achieve memory functionality. (Figure 9c) The programming process involves applying a light pulse and a negative gate pulse, which generate electron–hole pairs in the WSe_2_ channel. The resulting holes are trapped at the h‐BN/Al_2_O_3_ interface, altering the channel conductance. This state is retained as long as the trapped charges are not released, thus enabling nonvolatile memory. The erase process involves a light pulse and a positive gate pulse, which release the trapped charges, restoring the original conductance. This method provides robust endurance and long retention of the memory states. The synapse function in figure 9d is demonstrated by the ability to modulate the current states using different magnitudes and polarities of the gate voltage (*V*_R_). By applying sequences of light and electrical pulses, the phototransistor can achieve both long‐term potentiation (LTP) and long‐term depression (LTD), mimicking the behavior of biological synapses. The device shows stepwise increases and decreases in current, representing the strengthening and weakening of synaptic connections. This modulation is crucial for implementing learning and memory functions in neuromorphic computing applications.

Sahu et al.^[^ ^84^ ^]^ demonstrated that reconfigurable logic capable of performing both AND and OR gates could be achieved by exploiting the paired‐pulse facilitation characteristics of MoS_2_ transistors. The initial state acts as an AND gate, with the output defined as the current value that comes out after taking blue light and gate voltage as a single input (Figure 9e). When trained with UV pulses, it was shown that the logic function switches from AND to OR gate depending on the number of exposures or exposure time and returns to the initial state when a negative gate voltage is applied, demonstrating its reconfigurable characteristics. In addition, reconfigurable AND–OR switching logic gates have been developed using scandium nitride‐based transistors and dinaphtho[2,3‐b:2′,3′‐f]thieno[3,2‐b]thiophene/diarylethene (DNTT/DAE) bilayer‐based transistors. The synaptic function in the 2D MoS_2_ optoelectronic artificial synapse, as demonstrated in Figure 9f, showcases both LTP and LTD behaviors, essential for mimicking biological synapses. LTP is achieved by increasing the postsynaptic current (PSC) through repetitive blue optical pulses, indicating the strengthening of synaptic connections. Conversely, LTD is demonstrated by applying electrical gate pulses, which decrease the PSC, representing the weakening of synaptic connections. This dual modulation of synaptic weights allows the device to emulate the dynamic learning and memory functions of biological synapses, crucial for neuromorphic computing applications. The ability to control the PSC through optical and electrical stimuli provides a robust platform for developing in‐memory computing systems.

Li et al. studied α‐In_2_Se_3_ ferroelectric semiconductor, which provides both memory and synaptic functionalities.^[^ ^85^ ^]^ With a current on/off ratio of over 10^6^ and a high field‐effect mobility of 137.55 cm^2^ V^−1^ s^−1^, this device ensures rapid charge transport necessary to support memory and synaptic functions. Particularly, its high photosensitivity (98 mA W^−1^) contributes to storing and processing photon information. By modulating the gate voltage according to the optical signal, it can handle various spectra of visual information, providing a mechanism similar to synaptic weight adjustment. For instance, adjusting the gate voltage allows modulation of synaptic weights by changing the ferroelectric domain states in response to photon signals. This enables storage and reuse of visual information akin to synaptic plasticity. Through these mechanisms, the multifunctional semiconductor device processes visual information akin to neural circuits in the brain, enabling storage and reuse as needed. Such capabilities hold significant potential for application in next‐generation artificial intelligence and neuromorphic computing systems.

Liu et al. demonstrated how channel surface control in MoS_2_ enables logic gate operations and data storage. The thickness of MoS_2_ allows for adjustable logic functions, with separate gates controlling the top and bottom surfaces for OR and AND operations. Graphene is used as a trap layer for real‐time memory, integrating logic computing and data storage in a single 2D transistor cell. The FG between the surfaces stores charge, altering the channel's electrical characteristics to represent data. This mechanism enables simultaneous logic and memory functions within a single transistor.^[^ ^86^ ^]^

The retention time of the charge trapping mechanism, which is fundamental to n‐vr OELGs, currently lasts only several to tens of minutes. This limitation prevents the full realization of their nonvolatile potential. Additionally, similar to volatile reconfigurable logic devices, n‐vr OELGs face challenges such as delays caused by high resistive‐capacitive values and device size constraints due to lithography technology limitations. Addressing these issues is crucial for advancing the practical application and performance of n‐vr OELGs.

## 6 Discussion and Conclusion

The advancement of OELGs represents a significant leap in computational technology, merging optical and electronic components to enhance processing speed and energy efficiency. This review has explored the evolution of OELG architectures from 1D‐1L systems to more complex MD‐ML and 1D‐MLs, highlighting their potential and the challenges that need to be addressed to fully realize their capabilities.

### 6.1 Key Summary and Insights

#### 6.1.1 Single OELGs for Single‐Logic Operation (1D‐1L)

Initial research focused on unipolar photoresponsive photodetectors, which primarily implemented basic logic operations like OR and AND gates. However, limitations in controlling the current flow and ensuring the simultaneity of inputs posed significant challenges. Materials such as Si, polymers, vdW materials, and perovskites have been utilized, but more sophisticated designs are needed to perform complex logic operations.

#### 6.1.2 OELGs‐in‐Circuit for MD‐ML

Second‐generation OELGs integrate multiple devices in specific circuit arrangements, allowing each OELG to function independently based on the light inputs. This configuration enhances control and provides flexibility for multifunctional logic operations. Despite these advancements, the fixed nature of the logic operations once the circuit is fabricated and potential interference from binary inputs pose challenges for future downsized chips.

#### 6.1.3 Reconfigurable OELGs (1D‐ML)

Reconfigurable OELGs, similar to field‐programmable gate arrays, offer dynamic logic operation adjustments using additional stimuli like electric fields and light. These systems promise higher space efficiency and versatility, crucial for future applications in cognitive computing and visual information processing. Innovations such as bipolar photoresponse and OGMs have enabled successful implementation of multiple logic gates within a single device. However, improving the retention time, speed of state reconstruction, and minimizing power consumption are critical challenges.

#### 6.1.4 Nonvolatile OELGs

n‐vr OELGs and LIM devices integrate data storage with logic operations. LIM devices utilize FGs or charge trapping mechanisms for multibit states, suitable for in‐memory computing and edge devices. N‐vr OELGs emphasize reconfigurable logical functions, with recent studies demonstrating the potential for performing complex logic operations using heterostructure phototransistors.

While significant progress has been made in the development of OELGs, the following challenges must be addressed in order to fully realize the potential of OELGs.

### 6.2 Potential Applications

The emergence of OELGs has enabled novel applications beyond traditional sensing or computing platforms. Conventional imaging systems process data in two steps: preprocessing, which derives clear image data by excluding noise, and postprocessing, which identifies objects based on aritificial intelligence algorithms. OELGs have revolutionized the preprocessing unit. Kim's group fabricated a MoS_2_ transistor with a pV3D3 dielectric layer that demonstrates quasilinear synaptic plasticity changes depending on the number of exposed light pulses.^[^ ^87^ ^]^ Photogenerated charges are trapped at the MoS_2_/pV3D3 interface, resulting in excellent LTP. Utilizing this device, the research team devised a novel preprocessed imaging system that creates clear vision images without the need for additional memory, computing, or communication units. Das’ group expanded the preprocessing capability to develop an ultralow‐collision detector, a novel and practical application.^[^ ^88^ ^]^ They fabricated a MoS_2_ phototransistor stacked on a nonvolatile and programmable floating‐gate device architecture. Depending on the applied back‐gate voltage, the photocurrent pattern decreases as an object approaches the detector, reaching a local minimum just before a collision. This characteristic allows the device to predict the velocity and direction of an object without supplementary computing or memory units. Furthermore, preprocessing units based on photonic neural network hardware have been utilized in a wide range of vision systems, including broadband convolutional processing^[^ ^89^ ^]^ and pattern recognition.^[^ ^90^ ^]^

A key application of OELG devices is in hardware neural network processing. In particular, backpropagation algorithm is a method used in artificial neural networks to minimize the error by adjusting the weights through gradient descent, where the error is propagated backward from the output layer to the input layer. It calculates the gradient of the loss function with respect to each synaptic weight by the chain rule, updating the weights iteratively to reduce the error. The most common application of the backpropagation algorithm is the recognition of visual information, distinguishing objects or classes from photographic images. OELGs have been noted for their efficiency in reducing the required computing resources and power consumption by unifying the roles of image sensors and processing units into a single device. Park's group developed a neuromorphic device based on an h‐BN/WSe2 heterostructure, whose conductance can be linearly manipulated.^[^ ^90^ ^]^ They utilized the conductivity as a synaptic weight in a backpropagation algorithm and applied it to solve a sophisticated MNIST test with color‐mixed patterns. This device proved to be much more efficient in simultaneously classifying numbers and colors, surpassing the capabilities of conventional neural network systems without integrated vision sensors. Furthermore, neural processing units based on OELGs have been employed to address complex vision problems, such as sharpness or edge enhancement, and for preprocessing to obtain clear images.^[^ ^87^, ^88^, ^89^, ^90^ ^]^

### 6.3 Challenges and Future Directions

Next‐generation OELGs require new materials and device structures. For commercialization, it is essential to secure high endurance characteristics with over 10 m cycles and minimal performance degradation of less than one order of magnitude before fatigue. Optical devices are sensitive to environmental factors such as temperature and pressure, and short‐wavelength light can degrade performance and affect reliability. This necessitates the development of robust materials and protective layers. For n‐vr OELGs based on 2D materials, it is necessary to secure a retention time of over 10^4^ s and improve the operation speed to less than 10^−3 ^s.

Furthermore, integrating existing electronic devices with OELGs is essential. Developing structures like optical fibers that can deliver light to local areas while minimizing interconnections and interference between integrated chips is necessary. Improving the efficiency of OELGs while minimizing power consumption will be a key research goal, requiring the design and optimization of material properties and device architecture.

Unlike electronic logic chips, optical components pose a significant drawback in OELG systems. LEDs and other light sources are necessary in the hardware but are subject to optical diffraction limits, which hinder the miniaturization of the overall system. To address this issue, the development of a novel optical platform is required. Kim's group demonstrated an innovative OELG system capable of overcoming the optical diffraction limit, potentially revolutionizing the field.^[^ ^91^ ^]^ They proposed a vertically stackable chipset comprising micro‐LEDs and photodetectors at each pixel, functioning as data transmitters and receptors, respectively. Light emitted from an LED in the underlying chip reaches the upper chip layer at the exact same location, enabling chip‐to‐chip communication without the constraints of the optical diffraction limit. Despite this advancement, challenges remain in further miniaturizing the LED/photodetector pixels, reducing the power consumption of the light source, and lowering overall costs.

Similar to other electronic devices, OELG chips face significant challenges regarding compatibility with conventional Si complementary metal‐oxide‐semiconductor (CMOS) processes necessary for full commercialization. Most organic and organic–inorganic hybrid materials are severely damaged during the high‐temperature processes used in CMOS fabrication. Additionally, thin films with poor adhesion to a Si wafer are prone to peeling and contamination during conventional lithography or etching processes. Consequently, only commercial inorganic semiconductors such as Si or GaAs are selectively utilized in large‐area and dense OELG chips; however, they ultimately exhibit limited functionality and poor photosensitivity.^[^ ^92^, ^93^, ^94^, ^95^ ^]^

Advancements in transfer and lithography protocols have led to significant progress in the integration of 2D materials with Si waveguides over recent decades.^[^ ^96^, ^97^, ^98^, ^99^ ^]^ For example, Muller's group developed a CMOS‐compatible graphene/Si photodetector with a bandwidth exceeding 1 GHz.^[^ ^96^ ^]^ Similarly, a wide range of TMDCs have been utilized as key building blocks for CMOS‐compatible and high‐performance photodetectors, facilitating their incorporation into comparable OELGs.^[^ ^97^, ^98^, ^99^ ^]^ Despite numerous efforts to address CMOS compatibility, material scientists are yet to establish reliable methods for growing and processing wafer‐scale 2D materials. Furthermore, the development of organic or organic–inorganic semiconductors for integration into Si‐CMOS‐compatible hardware remains in its infancy, despite its critical importance for achieving high‐performance, cost‐effective OELGs with superior form factors.

In conclusion, despite current technical limitations, research and technological advancements in OELGs are progressing rapidly. Light is expected to play a crucial role in next‐generation logic devices, driving innovations that could transform computational technology. Addressing the outlined challenges will be essential to fully realize the potential of OELGs, paving the way for their application in high‐speed operation (over 10 GHz), low‐power consumption (under 100 nW), in‐memory computing, neuromorphic computing, and beyond.

## Conflict of Interest

The authors declare no conflict of interest.

## Author Contributions

**Woochul Kim**: Conceptualization (lead); Writing—original draft (lead). **Dante Ahn**: Conceptualization (equal); Software (lead); Writing—original draft (equal). **Minz Lee**: Software (supporting); Visualization (lead). **Namsoo Lim**: Software (supporting); Visualization (supporting). **Hyeonghun Kim**: Conceptualization (supporting); Writing—review & editing (equal). **Yusin Pak**: Conceptualization (equal); Writing—original draft (lead); Writing—review & editing (lead). Woochul Kim and Dante Ahn contributed equally to this work.
